# A Novel Antimicrobial Peptide Sparamosin_26–54_ From the Mud Crab *Scylla paramamosain* Showing Potent Antifungal Activity Against *Cryptococcus neoformans*

**DOI:** 10.3389/fmicb.2021.746006

**Published:** 2021-10-08

**Authors:** Yan-Chao Chen, Ying Yang, Chang Zhang, Hui-Yun Chen, Fangyi Chen, Ke-Jian Wang

**Affiliations:** ^1^State Key Laboratory of Marine Environmental Science, College of Ocean and Earth Sciences, Xiamen University, Xiamen, China; ^2^State-Province Joint Engineering Laboratory of Marine Bioproducts and Technology, College of Ocean and Earth Sciences, Xiamen University, Xiamen, China; ^3^Fujian Innovation Research Institute for Marine Biological Antimicrobial Peptide Industrial Technology, College of Ocean and Earth Sciences, Xiamen University, Xiamen, China

**Keywords:** antimicrobial peptide, Sparamosin_26–54_, fungicidal effect, membrane permeability, apoptosis

## Abstract

Due to the increasing prevalence of drug-resistant fungi and the limitations of current treatment strategies to fungal infections, exploration and development of new antifungal drugs or substituents are necessary. In the study, a novel antimicrobial peptide, named Sparamosin, was identified in the mud crab *Scylla paramamosain*, which contains a signal peptide of 22 amino acids and a mature peptide of 54 amino acids. The antimicrobial activity of its synthetic mature peptide and two truncated peptides (Sparamosin_1–25_ and Sparamosin_26–54_) were determined. The results showed that Sparamosin_26–54_ had the strongest activity against a variety of Gram-negative bacteria, Gram-positive bacteria and fungi, in particular had rapid fungicidal kinetics (killed 99% *Cryptococcus neoformans* within 10 min) and had potent anti-biofilm activity against *C. neoformans*, but had no cytotoxic effect on mammalian cells. The RNA-seq results showed that after Sparamosin_26–54_ treatment, the expression of genes involved in cell wall component biosynthesis, cell wall integrity signaling pathway, anti-oxidative stress, apoptosis and DNA repair were significantly up-regulated, indicating that Sparamosin_26–54_ might disrupt the cell wall of *C. neoformans*, causing oxidative stress, DNA damage and cell apoptosis. The underlying mechanism was further confirmed. Sparamosin_26–54_ could bind to several phospholipids in the cell membrane and effectively killed *C. neoformans* through disrupting the integrity of the cell wall and cell membrane observed by electron microscope and staining assay. In addition, it was found that the accumulation of reactive oxygen species (ROS) increased, the mitochondrial membrane potential (MMP) was disrupted, and DNA fragmentation was induced after Sparamosin_26–54_ treatment, which are all hallmarks of apoptosis. Taken together, Sparamosin_26–54_ has a good application prospect as an effective antimicrobial agent, especially for *C. neoformans* infections.

## Introduction

In addition to the serious threats caused by various virus outbreaks and drug-resistant bacterial infections, fungal infections are also similar headlines today. It is estimated that more than 1 billion people worldwide suffer from fungal diseases (Felix et al., 2017). Most fungal diseases are superficial infections of the skin and nails and mucosal infections of the oral cavity and genital tract, which are readily to diagnose and treat (Brown et al., 2012; Denning and Bromley, 2015). Invasive fungal infections mainly occur in immunocompromised patients, such as those infected with HIV or undergoing chemotherapy, which is associated with unacceptably high mortality (Miceli et al., 2011). It is estimated that nearly 2 million people die from fungal infections each year, and the death toll continues to rise (Pappas et al., 2010; Brown et al., 2012). About 90% of these deaths are caused by species belonging to one of the following four genera: *Cryptococcus*, *Candida*, *Aspergillus*, and *Pneumocystis*. *C. neoformans* is an opportunistic pathogen that exists widely in the environment and is one of the main species causing life-threatening cryptococcal meningitis. Globally, an estimated 223,100 cases of cryptococcal meningitis occur each year, resulting in 181,100 deaths (Rajasingham et al., 2017). Most deaths are reported to occur in low- and middle-income countries due to the lack of essential medicines and the high cost of effective treatment (Loyse et al., 2019).

In the past decades, a series of topical drugs have been used and moderate success has been achieved in the control of many superficial and mucosal infections. However, few drugs are available for invasive fungal infections (Perfect, 2017). Antifungal drugs are more difficult to develop than antibacterial drugs, because fungi are eukaryotes and have a high degree of similarity with mammalian cells, which means that there are relatively few differential targets to be exploited for antifungal drug development (Denning and Bromley, 2015). Currently, only four types of antifungal drugs (polyenes, flucytosine, azoles, and echinocandins) are used orally or intravenously for the treatment of invasive fungal infections in humans (Perfect, 2017). Although existing antifungal drugs are clinically useful, they do have some limitations, such as the development of drug resistance, undesirable side effects and host toxicity. The best example is the polyene antibiotic, amphotericin B, which was first isolated from the fermenter cultures of *Streptomyces nodosus* in 1959 (Dutcher et al., 1959). Amphotericin B has a relatively broad spectrum of action, and is still the drug of choice against serious infections with fungi such as *C. neoformans*, *Candida albicans*, or *Aspergillus fumigatus* (Lemke et al., 2005; Iyer et al., 2021). Like other polyene antibiotics, amphotericin B preferentially binds to ergosterol, which is the main sterol in fungal cell membranes, resulting in the disruption of cell membrane integrity (Campoy and Adrio, 2017). However, fungi can develop resistance to amphotericin B by reducing the content of ergosterol in the cell membrane. In recent years, some amphotericin B-resistant strains have been reported, including *C. neoformans*, *C. albicans*, and *A. fumigatus* (Ellis, 2002; Perlin et al., 2017). In addition, the adverse effects of amphotericin B are common, with nephrotoxicity being the most serious side effect (Fanos and Cataldi, 2000). Therefore, new antifungal agents are urgently needed to address the global threats of fungal pathogens and the limitations of existing antifungal drugs.

Antimicrobial peptides (AMPs) are considered to be natural antibiotics, which can be found in animals, plants and microorganisms, and function as the first line of host defense against the invasion of exogenous pathogen (Zasloff, 2002; Peschel and Sahl, 2006). They exert broad spectrum of activity against Gram-negative and Gram-positive bacteria, fungi, parasites, viruses and even antibiotic-resistant strains. The typical mechanism of AMPs action is to interact with cell membrane of microorganism through electrostatic interactions, causing membrane disruption, cytoplasmic leakage, and ultimately cell death. The proposed membrane disruption models include barrel-stave model, carpet model and toroidal-pore model (Brogden, 2005). Some AMPs also interfere with many important cellular functions, such as inhibition of cell wall synthesis, inhibition of nucleic-acid synthesis, inhibition of protein synthesis, inhibition of septum formation and inhibition of enzymatic activity (Brogden, 2005). Interestingly, the antimicrobial mechanism of AMPs is different from most clinically antibiotics that only target cell membrane/wall components (Rautenbach et al., 2016). This interactions with the fundamental physiological structure and multi-target modes make it difficult for microorganisms to develop resistance (Yeung et al., 2011), making AMPs the most promising potential alternative for conventional antibiotics.

Crustaceans are a group of invertebrates that only rely on the innate immune system to defend them against invading pathogenic microorganisms. The innate immune system of crustaceans is comprised of cellular and humoral responses. In terms of humoral responses, AMPs are one of the main effectors to eliminate pathogenic microorganisms (Rowley and Powell, 2007). There is clear evidence that many AMPs with potent antibacterial activity have been isolated from crustaceans, such as anti-lipopolysaccharide factors (ALFs), Hyastatin, scygonadin, Sphistin, etc. (Wang et al., 2007; Sperstad et al., 2009b; Liu et al., 2012; Chen et al., 2015). However, the number of AMPs with effective activity against fungi is reported to be very limited. Penaeidins, a family of cationic AMPs were isolated from shrimps, such as *Litopenaeus vannamei* (Destoumieux et al., 1997; Gueguen et al., 2006). The recombinant penaeidins (Pen-2 and Pen-3a) exhibit not only antibacterial activity against Gram-positive bacteria, but also antifungal activity against several filamentous fungi (including *Fusarium oxysporum*, which caused infections in penaeid shrimps) (Destoumieux et al., 1999; Shockey et al., 2009). Crustins are another well-studied AMPs with antifungal activity in crustaceans, which are cationic and cysteine-rich AMPs containing a whey acidic protein (WAP) domain in the C-terminus (Smith et al., 2008). A crustin gene, Cru*Ha*1 from the spider crab *Hyas araneus*, had moderate activity against *S. cerevisiae* and weak activity against *C. albicans* (Sperstad et al., 2009a). Several AMPs currently identified from crustaceans have antibacterial activity as well as antifungal activity, however, their antifungal mechanism has not yet been revealed. In addition, there are more new antifungal peptides from crustaceans waiting to be discovered and studied.

In this study, we screened a new antimicrobial peptide based on the transcriptome database of the mud crab *S*. *paramamosain* established in our laboratory, and named it Sparamosin. The full-length cDNA sequence of Sparamosin gene was obtained. Sparamosin mature peptide and its two truncated peptides (Sparamosin_1–25_ and Sparamosin_26–54_) were chemically synthesized, and their *in vitro* antimicrobial activity were determined. Among these three peptides, Sparamosin_26–54_ showed the strongest antimicrobial activity, which was used to carry out a series of follow-up studies, such as evaluating its anti-biofilm activity. To investigate the antifungal mechanism of Sparamosin_26–54_ against *C. neoformans*, RNA-seq was used to identify the differentially expressed genes and the pathways involved. Confocal laser scanning microscopy, scanning electron microscope and transmission electron microscope were used to provide further evidence on the antifungal mechanism of Sparamosin_26–54_ against *C. neoformans*. This study will provide basic information for the development of new antifungal drugs.

## Materials and Methods

### Animals and Microorganisms

Mud crabs (*S. paramamosain*) were obtained from Zhangpu Fish Farm (Fujian, China), and testes from male crabs (body weight 300 ± 10 g) were used for RNA extraction. All commercially available strains used in this study were purchased from China General Microbiological Culture Collection Center (CGMCC), including *Pseudomonas fluorescens* (CGMCC NO. 1.3202), *Pseudomonas stutzeri* (CGMCC NO. 1.1803), *Pseudomonas aeruginosa* (CGMCC NO. 1.2421), *Acinetobacter baumannii* (CGMCC NO. 1.6769), *Escherichia coli* (CGMCC NO. 1.2385), *Staphylococcus aureus* (CGMCC NO. 1.2465), *Staphylococcus epidermidis* (CGMCC NO. 1.4260), *Bacillus cereus* (CGMCC NO. 1.3760), *Enterococcus faecium* (CGMCC NO. 1.131), *Enterococcus faecalis* (CGMCC NO. 1.2135), *C. neoformans* (CGMCC NO. 2.1563), *Aspergillus niger* (CGMCC NO. 3.316), *A. fumigatus* (CGMCC NO. 3.5835), *Fusarium graminearum* (CGMCC NO. 3.4521), and *F. oxysporum* (CGMCC NO. 3.6785). The bacterial strains were cultured in nutrient broth at 37°C, and the fungal strains were grown in potato dextrose agar (PDA) at 28°C. All experiments were carried out in strict accordance with the guidelines of the standard biosecurity and institutional safety procedures established by Xiamen University.

### Cloning the Full-Length cDNA of Sparamosin

Rapid amplification of cDNA ends (RACE) PCR was performed to obtain the full-length cDNA of Sparamosin. Total RNA from the testes of normal mature crabs was extracted using TRIzol^TM^ reagent (Invitrogen, United States) and cDNA was synthesized using PrimeScript^TM^ RT reagent Kit with a gDNA Eraser Kit (Takara, China). In addition, RACE cDNA was prepared using SMARTer^®^ RACE 5′/3′ Kit (Takara, China) and was used as a template for PCR. Gene-specific primers (Supplementary Table 1) were designed based on the obtained partial cDNA sequence from transcriptome database established by our laboratory to amplify the target gene, and the fragment was recombined into pMD18-T Vector (Takara, China) and sequenced by Bioray biotechnology (Xiamen, China).

### Sequence Analysis, Peptides Design, and Synthesis

The signal peptide of Sparamosin was predicted by SignalP-4.1 Server^1^ and the second structure of the mature peptide was predicted by PSIPRED 4.0.^2^ The mature peptide was truncated between residue Ser^25^ and residue Gly^26^ to evaluate the antimicrobial activity of different helical regions. The physicochemical parameters of the peptides, such as molecular weight, theoretical isoelectric point, net positive charge and hydropathicity were predicted by ProtParam.^3^ The total hydrophobicity was calculated through the Antimicrobial Peptide Database.^4^ The peptides used in this study were chemically synthesized by Genscript (Nanjing, China). The purity and molecular weight of the peptides were further confirmed by high performance liquid chromatography and mass spectrometry, respectively. The powdered peptide could be stored for a long time at −80°C. The stock solutions were kept at −20°C for storage.

### Antimicrobial Activity Assay

The antimicrobial activity was determined three times, each time in triplicate, in 96-well microplates according to previous reports with some modifications (Vitale et al., 2002; Wiegand et al., 2008; Castro et al., 2018). Briefly, microorganisms were harvested during their logarithmic growth phase and diluted in Mueller-Hinton broth to approximately 1 × 10^6^ CFU/mL (bacteria) or diluted in RPMI 1640 medium buffered with 0.165 mol/L 3-morpholinopropane-1-sulfonic acid (pH 7.0, referred to as RPMI-MOPS) to approximately 2 × 10^4^ cells/mL (yeasts or conidia of filamentous fungi). Then, the microbial suspension was added to each well and incubated with serial diluted peptides. The microplates were incubated in the dark at 37°C for 24 h (bacteria) or at 28°C for 48 h (yeasts or conidia of filamentous fungi). The minimum inhibitory concentration (MIC), minimum bactericidal concentration (MBC), and minimum fungicidal concentration (MFC) values were determined as previously described (Shan et al., 2016).

### Cytotoxicity Assay

The cytotoxicity of Sparamosin_26–54_ was determined on mouse hepatocytes (AML 12 cells) and human hepatocytes (L02 cells). CellTiter 96^®^ AQ_*ueous*_ one solution Cell Proliferation assay (Promega, United States) was used to assess cell viability. Briefly, 100 μL of AML 12 or L02 cells were seeded in 96-well microplates at 10^4^ cells/well and incubated at 37°C overnight under 5% CO_2_. Then, the cells were incubated at 37°C for 24 h in a culture medium supplemented with various concentrations of Sparamosin_26–54_ (0, 0.1, 1, 10, 100 μg/mL). After incubation, the cells were treated with 20 μL of MTS-PMS reagent for another 2 h, and then the absorbance value of each well was measured at 492 nm (Tecan, Switzerland).

### Biofilm Inhibition Assays

The biofilm inhibition assays were performed in 96-well microplates according to previous reports with some modifications (Berditsch et al., 2015; Poonam et al., 2017). For the biofilm formation assay, *C. neoformans* cells were harvested in the logarithmic phase, diluted in RPMI-MOPS medium to a final cell density of approximately 1 × 10^6^ cells/mL, and then aliquoted into microplates. Serially diluted concentrations of Sparamosin_26–54_ were added to the wells, and the microplates were incubated at 35°C for 72 h without shaking to allow biofilm formation. The biofilm mass was evaluated by crystal violet (CV) staining as previously described (Berditsch et al., 2015). In experiments with preformed biofilms, *C. neoformans* cells were incubated at 35°C for 72 h without shaking to allow biofilm formation. Thereafter, RPMI-MOPS medium containing Sparamosin_26–54_ and resazurin (final concentration 0.1 mM) was added to the wells, and the microplates were incubated at 35°C for another 24 h. The respiratory activity of cells in biofilm was evaluated by a modified resazurin assay as previously described (Berditsch et al., 2015).

### Transcriptome Analysis of *C. neoformans* After Sparamosin_26–54_ Treatment

*C*. *neoformans* suspensions at approximately 1 × 10^7^ cells/mL in RPMI-MOPS were incubated with different concentrations of Sparamosin_26–54_ (6, 12 μM) at 28°C for 1 h. Then, the cells were harvested for RNA extraction. Approximately 200 μL of freeze-dried cells were grinded in liquid nitrogen and the total RNA was isolated using TRIzol reagent. RNA-Seq was performed by Novogene Corporation (Beijing, China) using the Illumina NovaSeq platform, which could generate 150 bp paired-end reads. Reference genome and gene model annotation files of *C*. *neoformans* var. *neoformans* JEC21 were downloaded from GenBank (GCA_000091045.1). An index of the reference genome was constructed using Hisat2 (version 2.0.5), and paired-end clean reads were aligned with the reference genome using Hisat2 (version 2.0.5). FeatureCounts (version 1.5.0-p3) was used to calculate the number of reads mapped to each gene. The expected number of fragments per kilobase of transcript sequence per million base pairs sequenced (FPKM) for each gene was calculated based on the length of the gene and the count of reads mapped to that gene. Differential expression analysis of the two groups was performed using the DEGSeq2 R package (version 1.16.1). The *p*-value was adjusted using the Benjamini and Hochberg’s method. The corrected *p-*value < 0.05 and | log_2_(Fold change)| > 1 were set as thresholds for significant differential expression. The gene ontology (GO) enrichment analysis of differentially expressed genes (DEGs) was implemented by the clusterProfiler R package (version 3.4.4). GO terms with a corrected *p*-value < 0.05 were considered significantly enriched by DEGs.

To verify the results of RNA-Seq, five up-regulated genes encoding chitin synthase (CNA05300), Rho1 GTPase (CNG02630), catalase (CNL06020), DNA supercoiling (CND02890) and caspase (CNB00130) and six down-regulated genes encoding C-22 sterol desaturase (CNF03720), NADH dehydrogenase (CND01070), succinate dehydrogenase (CNA03530), ubiquinol-cytochrome *c* reductase complex core protein 2 (CNL04470), cytochrome *c* oxidase subunit V (CNK03240) and ATP synthase subunit alpha (CNF02280) were selected for quantitative real-time PCR (qRT-PCR). The qRT-PCR reactions were performed on qTOWER 2.2 real time PCR system (Analytik Jena AG, Germany) according to the FastStart Universal SYBR Green Master (ROX) kit (Roche, Switzerland) protocol. The cycling conditions were 50°C for 2 min, 95°C for 10 min, followed by 40 cycles of 95°C for 15 s, 60°C for 1 min. Gene expression levels were calculated using the 2-ΔΔ^Ct^ method (Livak and Schmittgen, 2001) and normalized to the abundance of the house-keeping gene *actin*. The primers of target genes were listed in Supplementary Table 2.

### Localization of Sparamosin_26–54_ in *C. neoformans*

The analysis of the localization of Sparamosin_26–54_ in *C. neoformans* was carried out based on the previous report with slight modifications (Zhang et al., 2019). Briefly, *C. neoformans* cells were harvested in the logarithmic phase and washed with 10 mM sodium phosphate buffer (NaPB, pH 7.4). The cells were diluted in the same buffer to a final cell density of approximately 1 × 10^7^ cells/mL, and incubated with 24 μM FITC-labeled Sparamosin_26–54_ [synthesized by GL Biochem (Shanghai, China)] at room temperature for 30 min. After incubation, the cells were washed twice with 10 mM NaPB to remove unbound peptide. A confocal laser scanning microscopy (Zeiss, Germany) were used for imaging.

### Protein-Phospholipid Interaction Assay

The polyclonal antibody of Sparamosin was prepared by GenScript (Nanjing, China) using the antigen site KVQHSIFSGLGPNPC, which was designed and optimized by OptimumAntigen Design Tool. The Sparamosin_26–54_-phospholipid interactions were determined using PIP Strips^TM^ (Echelon Biosciences, United States) according to the protocol. Briefly, the membrane was blocked with 3% (wt/vol) BSA at room temperature for 1 h, and then incubated with 2 μg/mL Sparamosin_26–54_ at room temperature for 1 h. Then, the membrane was incubated with Sparamosin antibody (1:1,000, diluted in 1% [wt/vol] BSA) at room temperature for 2 h. After washing with PBST several times, the membrane was then incubated with HRP conjugated goat anti-rabbit IgG (1:5,000, diluted in 1% [wt/vol] BSA) at room temperature for 40 min. The peptides were visualized on a chemiluminescent imaging system (Tanon Science and Technology, United States) using immobilon^TM^ western chemiluminescent HRP substrate (Millipore, United States).

### Time-Killing Kinetics

The time-killing kinetics assay was carried out as previous described (Shan et al., 2016). Briefly, *C. neoformans* cells were diluted in RPMI-MOPS medium to a final cell density of approximately 1 × 10^6^ cells/mL. Sparamosin_26–54_ was incubated with *C. neoformans* at a concentration of 1 × or 2 × MIC. The culture was sampled diluted and plated on PDA plates at different time points. After 48 h of incubation, the surviving colonies were counted, and the untreated group was used as a control. The experiments were conducted three times independently.

### Scanning Electron Microscope Analysis

The effect of Sparamosin_26–54_ on *C. neoformans* was observed using SEM as described earlier (Datta et al., 2016). Briefly, *C. neoformans* cells were harvested in the logarithmic phase and resuspended at approximately 1 × 10^7^ cells/mL in NaPB, and incubated with 24 μM Sparamosin_26–54_ at room temperature for 1 h. After incubation, the cells were fixed with 2.5% (vol/vol) glutaraldehyde at 4°C for 2 h and washed three times before being placed on poly-L-lysine coated glass slides at 4°C for 30 min. The cells were subsequently dehydrated in a graded series of ethanol (30, 50, 70, 90, 95, and 100%), for 15 min each. Thereafter, the samples were dehydrated with tertiary butanol followed by freezing at 4°C and lyophilized using the critical point dryer. Finally, the specimens were coated with gold and observed under a field emission scanning electron microscope (Zeiss SUPRA 55, Germany).

### Transmission Electron Microscope Analysis

The TEM analysis was carried out based on the previous report with slight modifications (Chen et al., 2003). Briefly, *C. neoformans* cells were prepared and incubated with Sparamosin_26–54_ as described above for SEM analysis. After pre-fixation with 2.5% glutaraldehyde at 4°C overnight, the *C. neoformans* cells were washed three times with NaPB and then post-fixed with 1% osmium tetroxide at 4°C for 2.5 h. The fixed samples were washed three times with NaPB, dehydrated in a graded ethanol series (30, 50, 70, 90, and 100%), transferred to a graded mixture of absolute acetone and epoxy resin, and finally immersed in pure epoxy resin in a constant-temperature incubator overnight. Finally, the samples were sectioned using an ultramicrotome, stained with uranyl acetate and lead citrate, and observed using a transmission electron microscope (FEI, United States).

### Live-Dead Staining Assay

The integrity of the cell membrane after Sparamosin_26–54_ treatment was determined by live-dead staining according to the protocol of a LIVE/DEAD^®^ FungaLight^TM^ Yeast Viability Kit (Invitrogen, United States). Briefly, *C. neoformans* cells were prepared and incubated with Sparamosin_26–54_ as described above for SEM analysis. After incubation, the cells were washed twice with PBS and placed on poly-L-lysine slides at room temperature for 15 min. The cells were washed twice and then stained with SYTO^®^ 9 and PI for 15 min in the dark. Fluorescent images were obtained with a confocal laser scanning microscopy (Zeiss, Germany).

### Calcein Leakage Assay

The calcein leakage assay was carried out according to a previous report with the following modifications (Vylkova et al., 2007). Briefly, *C. neoformans* cells were harvested in the logarithmic phase and washed with 10 mM NaPB. The cells were diluted in 10 mM NaPB to a final cell density of approximately 1 × 10^7^ cells/mL and loaded with Calcein-AM (Thermo Fisher Scientific, United States) at a final concentration of 5 μM at room temperature for 2 h. Then, the cells were washed three times with 10 mM NaPB to remove unincorporated dye. Different concentrations of Sparamosin_26–54_ (from 6 to 48 μM) were incubated with calcein-loaded cells in a 96-well microplate for analysis. In a microplate reader (Tecan, Switzerland), the fluorescence intensity of the induced calcein release was recorded every 15 min at the excitation and emission wavelengths of 485 nm and 530 nm, respectively. Percent leakage was calculated using the formula: percentage leakage (%) = (F_*sample*_ − F_0_)/(F_*T*_ − F_0_) × 100. Where F_*sample*_ is the fluorescence intensity after adding Sparamosin_26–54_, and F_0_ is the fluorescence intensity determined by measuring the amount of calcein released from loaded cells without peptide treatment in 240 min, and F_*T*_ is the maximum fluorescence intensity measured after the cells were boiled for 10 min. After boiling, the fluorescence intensity of calcein-loaded *C*. *neoformans* was assumed to be equal to the total potentially available intracellular calcein.

### DNA and ATP Release Assay

*C*. *neoformans* suspensions at 1 × 10^7^ cells/mL in RPMI-MOPS were incubated with different concentrations of Sparamosin_26–54_ (6, 12, and 24 μM) at 28°C for 0.5, 1, and 2 h. Then, the yeasts were collected by centrifugation, and the supernatant was subjected to DNA quantitative analysis in a NanoDrop 2000 spectrophotometer (Thermo Fisher Scientific, United States), or an ATP determination kit (Invitrogen, United States) for quantitative analysis of ATP, as described previously (Castro et al., 2018; Liu et al., 2020).

### Apoptosis Analysis

The production of intracellular reactive oxygen species (ROS) was measured by fluorometric assay using 2,7-dichlorofuorescin diacetate (DCFH-DA), as described previously (Hwang et al., 2011b). Briefly, *C*. *neoformans* suspensions at 1 × 10^7^ cells/mL in RPMI-MOPS were incubated with Sparamosin_26–54_ (6, 12 μM) or 20 mM H_2_O_2_ at 28°C for 1 h. After incubation, the cells were washed with PBS and then stained with 10 μM of DCFH-DA (Nanjing Jiancheng Bioengineering Institute, China). The cells were then harvested and observed under fluorescence microscopy (Zeiss, Germany).

The mitochondrial membrane potential (MMP) was determined by fluorometric assay using 3,3′-dihexyloxacarbocyanine iodide DiOC_6_(3), as mentioned earlier (Hwang et al., 2011b). Briefly, *C*. *neoformans* suspensions at 1 × 10^7^ cells/mL in RPMI-MOPS were incubated with Sparamosin_26–54_ (6, 12 μM) or 20 mM H_2_O_2_ at 28°C for 3 h. After incubation, the cells were washed with PBS, and incubated with 2 ng of DiOC_6_(3) (Sigma, United States) at 28°C for 30 min. The cells were then harvested and analyzed by flow cytometer (Becton Dickinson, United States).

Terminal deoxynucleotidyl transferase-mediated dUTP-nick end labeling (TUNEL) assay was carried out as described previously (Castro et al., 2018). Briefly, *C*. *neoformans* suspensions at 1 × 10^7^ cells/mL in RPMI-MOPS were incubated with Sparamosin_26–54_ (6, 12 μM) or amphotericin B (4 μg/mL) at 28°C for 24 h. After incubation, the cells were washed with PBS and fixed with 2% formaldehyde in PBS at room temperature for 30 min. Then, the cells were permeabilized with 0.1% Triton X-100 for 30 min and subjected to TUNEL reaction using one step TUNEL apoptosis assay kit (Beyotime, China), according to the manufacturer’s instructions. Finally, all samples were examined by fluorescence microscopy (Zeiss, Germany).

### Statistical Analysis

All data were represented as mean ± standard error of mean. Statistical analysis was performed with Student’s *t*-test using GraphPad Prism 6 (GraphPad Software Inc., United States) and SPSS 25 (IBM Corp., United States). Two-tailed *p*-values were used for all analyses, and a *p*-value < 0.05 was considered statistically significant. Transcriptome analysis was performed using Novomagic (Novogene, China). Pearson analysis was used to correlate gene expression determined by RNA-seq and qRT-PCR.

## Results

### Sequence Analysis and Truncated Peptides Design

**FIGURE 1:**
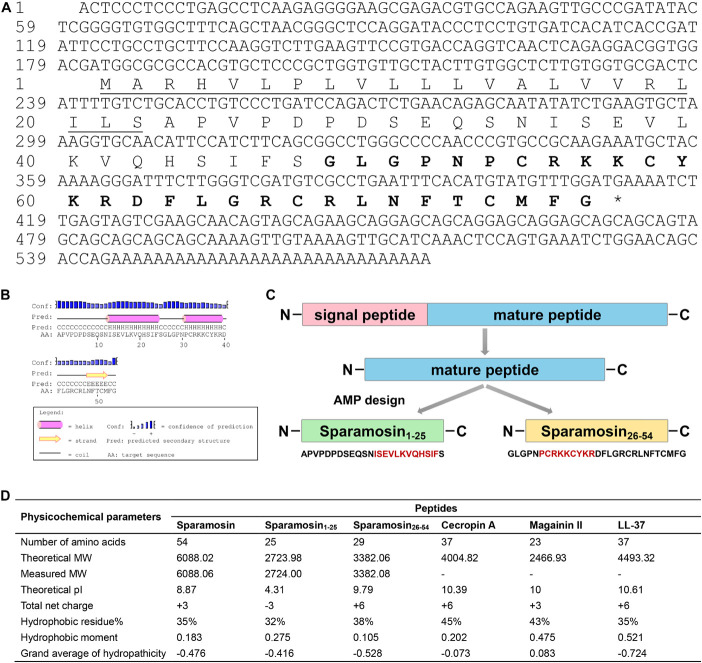
Sequence analysis and peptide design process. **(A)** Full-length cDNA and deduced amino acid sequence of Sparamosin. The underline indicates the putative signal peptide. The amino acid sequence of Sparamosin_26_***_–_***_54_ is shown in bold. The cDNA sequence has been deposited in GenBank and the accession number is MH423837. **(B)** The predicted secondary structure of Sparamosin mature peptide. There are two predicted α-helices located at residues Ile^13^-Phe^24^ and residues Pro^31^-Arg^39^, respectively. **(C)** The design process of truncated peptide. The red amino acid sequence represents the predicted α-helix structure. **(D)** The key physicochemical parameters of Sparamosin mature peptide and its truncated peptides, Cecropin A, Magainin II, and LL-37 were calculated by using online tools. Their molecular weight (MW) were measured by electrospray ionization mass spectrometry (ESI-MS).

The full-length cDNA sequence of Sparamosin was obtained (Genbank accession number MH423837). This gene consists of a 5′ untranslated region (UTR) of 181 bp, an open reading frame (ORF) of 231 bp and a 3′ UTR of 161 bp. The ORF of Sparamosin encodes a 76-amino acid protein, which contains a putative 22-amino acid signal peptide and a 54-amino acid mature peptide (Figure 1A). No similar nucleotide or amino acid sequence was found to match this sequence in the existing online databases, indicating that it is an uncharacterized protein. Two truncated peptides, Sparamosin_1–25_ and Sparamosin_26–54_ (which located in the 1st to 25th and 26th to 54th amino acid of the mature peptide, respectively), were designed and synthesized based on the secondary structure of Sparamosin mature peptide, which has two predicted α-helices located at residues Ile^13^-Phe^24^ and residues Pro^31^-Arg^39^, respectively (Figures 1B,C). The key physicochemical parameters of Sparamosin and its truncated peptides were shown in Figure 1D. In addition, the peptide sequences of Cecropin A (Steiner et al., 1981), Magainin II (Zasloff, 1987), and LL-37 (Gudmundsson et al., 1996) were also analyzed as comparative controls. The measured molecular weights of Sparamosin and its truncated peptides were consistent with their theoretical values, indicating that these peptides were successfully synthesized. The hydrophobic residue ratio of six peptides ranged from 32 to 45%, the net charge ranged from −3 to + 6, and the hydrophobic moment ranged from 0.105 to 0.521. The results showed that unlike the other two synthetic peptides (Sparamosin and Sparamosin_1–25_), the physicochemical parameters of Sparamosin_26–54_ have values closer to those defined for AMP.

### The Truncated Peptide Sparamosin_26–54_ Has Potent and Broad-Spectrum Antimicrobial Activity Without Cytotoxicity

Our preliminary study on six commercially available CGMCC strains showed that Sparamosin_26–54_ displayed stronger antimicrobial activity than Sparamosin, while no antimicrobial activity of Sparamosin_1–25_ was observed in the tested strains (Supplementary Table 3). Then, we further evaluated the antimicrobial efficacy of Sparamosin_26–54_ by determining its MIC, MBC and MFC against a series of strains. As shown in Table 1, Sparamosin_26–54_ displayed a broad-spectrum antibacterial activity against several Gram-negative (*P. fluorescens*, *P. stutzeri*, *P. fluorescens*, *A. baumannii*, *E. coli*) and Gram-positive (*S. aureus*, *S. epidermidis*, *B. cereus*, *E. faecium*, *E. faecalis*) bacteria with MIC values in the range of 6–48 μM, as well as the MBC values lower than 48 μM. In addition, Sparamosin_26–54_ showed a profound inhibitory effect against a pathogenic fungus *C. neoformans*, and could inhibit the conidial germination of several filamentous fungi, such as *A. niger*, *A. fumigatus*, *F. graminearum*, and *F. oxysporum* with MIC values in the range of 6–24 μM. However, it was not cytotoxic to mammalian cells tested (AML12 and L02 cells) (Supplementary Figures 1A,B), indicating that Sparamosin_26–54_ had good biocompatibility.

**TABLE 1:** Antimicrobial activity of synthetic Sparamosin_26–54_.

Microbial strains	CGMCC No.^*a*^	MIC^*b*^ (μM)	MBC^*c*^/MFC^*d*^ (μM)	MIC (μM)
**Gram-negative bacteria**		**Sparamosin_26–54_**		**LL-37**

*Pseudomonas fluorescens*	1.3202	6–12	12–24	3–6
*Pseudomonas stutzeri*	1.1803	6–12	12–24	3–6
*Pseudomonas aeruginosa*	1.2421	12–24	24–48	12–24
*Acinetobacter baumannii*	1.6769	12–24	12–24	3–6
*Escherichia coli*	1.2385	24–48	24–48	6–12

**Gram-positive bacteria**		**Sparamosin_26–54_**		**LL-37**

*Staphylococcus aureus*	1.2465	12–24	24–48	6–12
*Staphylococcus epidermidis*	1.4260	6–12	12–24	6–12
*Bacillus cereus*	1.3760	24–48	>48	12–24
*Enterococcus faecium*	1.131	6–12	6–12	3–6
*Enterococcus faecalis*	1.2135	12–24	12–24	3–6

**Fungi**		**Sparamosin_26–54_**		**Amphotericin B**

*Cryptococcus neoformans*	2.1563	6–12	12–24	0.12–0.24
*Aspergillus niger*	3.316	6–12	>48	0.12–0.24
*Aspergillus fumigatus*	3.5835	12–24	>48	0.24–0.48
*Fusarium graminearum*	3.4521	6–12	>48	0.12–0.24
*Fusarium oxysporum*	3.6785	6–12	>48	0.12–0.24

*^a^CGMCC NO., China General Microbiological Culture Collection Center Number. ^bcd^The values of MIC and MBC/MFC are expressed as the interval [a]-[b]. [a] is the highest concentration with visible microbial growth in the tested, and [b] is the lowest concentration with no visible microbial growth.*

### Sparamosin_26–54_ Has Anti-biofilm Activity Against *C. neoformans*

**FIGURE 2:**
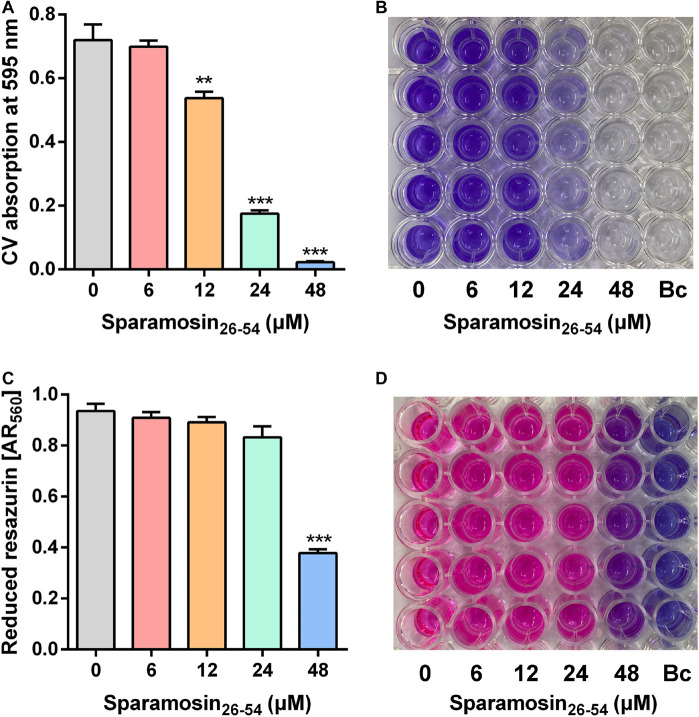
The inhibitory effect of Sparamosin_26_***_–_***_54_ against *C*. *neoformans* biofilm. **(A,B)** The inhibitory effect of Sparamosin_26_***_–_***_54_ on the formation of *C*. *neoformans* biofilm. The biofilm mass was quantified by CV staining, and the absorbance at 595 nm was measured. Bc, blank control. **(C,D)** The inhibitory effect of Sparamosin_26_***_–_***_54_ against the preformed biofilm of *C*. *neoformans*. The amount of reduced resazurin (resorufin) was determined by measuring the absorbance at 560 nm, and the residual amount of oxidized resazurin was quantified by measuring the absorbance at 620 nm. The corrected A_560_ value (AR_560_) was calculated using the following formula: AR_560_ = A_560_ − (A_620_ × R_*O*_) and R_*O*_ = AO_560_/AO_620_, where A_560_ and A_620_ are sample absorbance and AO_560_ and AO_620_ are the absorbance of RPMI-MOPS medium containing 0.1 mM resazurin. Bc, blank control. Data represent mean ± standard error of mean from three independent biological replicates. Significant difference between control group and Sparamosin_26_***_–_***_54_ treatment group was indicated by asterisks as **p < 0.01; ***p < 0.001.

Since Sparamosin_26–54_ showed good activity against planktonic *C. neoformans* cells, we further evaluated whether Sparamosin_26–54_ could inhibit *C. neoformans* biofilm formation. The biofilm mass of *C. neoformans* was quantified by CV staining, and the results were shown in Figures 2A,B. The concentration of Sparamosin_26–54_ required to inhibit *C. neoformans* biofilm formation was 12 μM. At a concentration of 48 μM, the inhibition rate of biofilm formation was more than 90%. In addition, we used the redox indicator resazurin to monitor the respiratory activity of the preformed *C. neoformans* biofilms treated with Sparamosin_26–54_. The results showed that Sparamosin_26–54_ could inhibit the respiration of *C. neoformans* in preformed biofilms. At a concentration of 48 μM, the inhibition rate of respiratory activity exceeded 50% (Figures 2C,D). These results suggested that Sparamosin_26–54_ exhibited potent anti-biofilm activity against *C. neoformans*.

### The Molecular Response of *C. neoformans* to Sparamosin_26–54_

**FIGURE 3:**
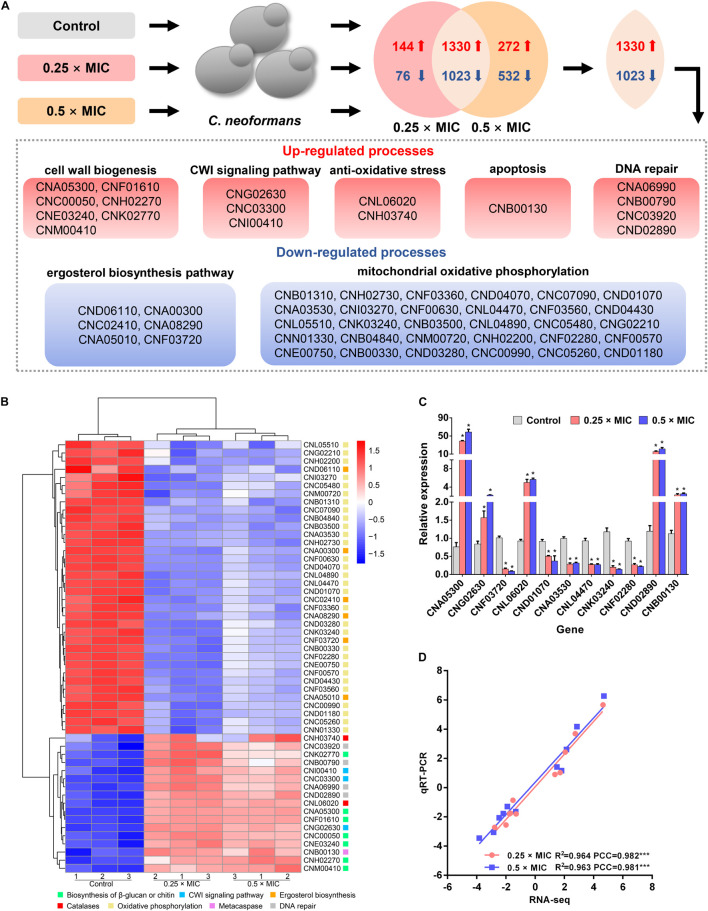
Transcriptomic analysis of Sparamosin_26_***_–_***_54_-treated *C. neoformans*. **(A)** A common transcriptional response to different concentrations of Sparamosin_26_***_–_***_54_ was identified by RNA-seq. **(B)** Heatmap of partial up-regulated and down-regulated genes. **(C)** Gene expression profiles of 11 DEGs in Sparamosin_26_***_–_***_54_-treated *C. neoformans*. Significant difference between control group and Sparamosin_26_***_–_***_54_ treatment group was indicated by asterisks as *p < 0.05. **(D)** Correlation analysis between qRT-PCR and RNA-Seq results. PCC, Pearson correlation coefficients. ***p < 0.001.

To build a comprehensive model of *C*. *neoformans* response to Sparamosin_26_***_–_***_54_, RNA sequencing was used to determine DEGs in response to treatment with a low dose of Sparamosin_26_***_–_***_54_. We obtained 2,573 genes (up-regulated, 1,474; down-regulated, 1,099) and 3,157 genes (up-regulated, 1,602; down-regulated, 1,555) that were differentially expressed after 0.25 × and 0.5 × MIC Sparamosin_26_***_–_***_54_ treatment, respectively (Supplementary Tables 4, 5). Overlapping analysis showed that 1,330 genes were commonly up-regulated under both Sparamosin_26_***_–_***_54_ concentrations tested, while 1,023 genes were commonly down-regulated under the same conditions (Figure 3A and Supplementary Tables 6, 7). To further investigate the biological processes involving these up-regulated or down-regulated genes, two DEG lists were separately analyzed for enrichment of GO terms. Genes up-regulated after Sparamosin_26_***_–_***_54_ treatment were associated with several significantly enriched biological processes, including cell wall organization or biogenesis, transmembrane transport, lipid oxidation, DNA repair, etc. (Supplementary Table 8). In the biological process category of the down-regulated genes, translation and metabolic processes were significantly enriched (Supplementary Table 8). Figure 3B is a heatmap showing the up-regulated genes involved in the biosynthesis of cell wall component, CWI signaling pathway, anti-oxidative stress, apoptosis and DNA repair, as well as the downregulated genes involved in the ergosterol biosynthesis pathway and mitochondrial oxidative phosphorylation in *C*. *neoformans* cells after Sparamosin_26_***_–_***_54_ treatment. To verify the RNA-Seq results, a total of 11 genes were selected including 5 up-regulated and 6 down-regulated from DGEs for qRT-PCR analysis. As shown in Figures 3C,D, the qRT-PCR results showed that the expression patterns of selected genes were consistent to those obtained by RNA-seq and the Pearson correlation analysis revealed that these genes were significantly correlated between the RT-qPCR and RNA-seq (Pearson correlation coefficient > 0.98). Thus, the qRT-PCR results confirmed the reliability of the RNA-Seq data.

### Sparamosin_26–54_ Exerts Its Antifungal Activity Through Targeting Fungal Membrane

**FIGURE 4:**
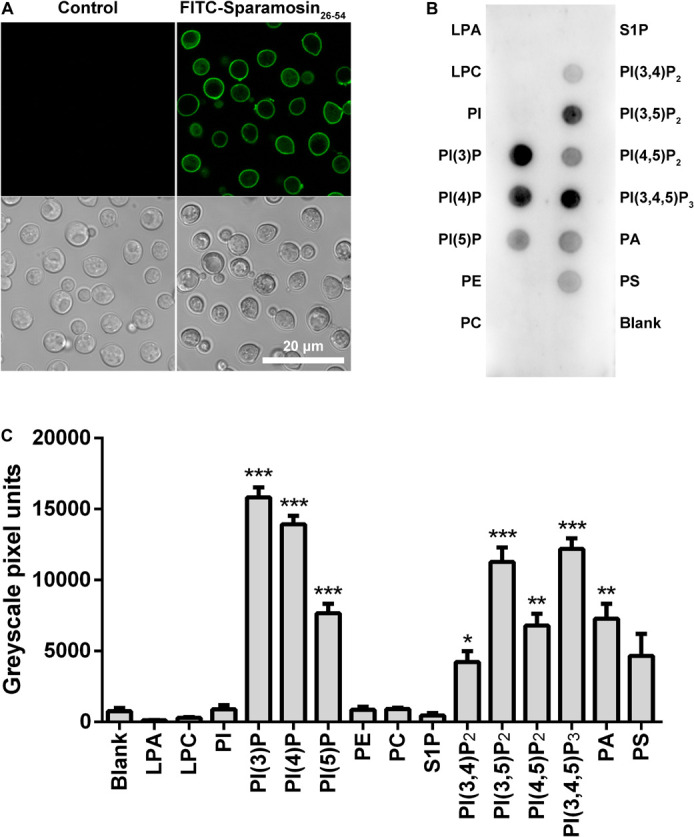
Localization of Sparamosin_26_***_–_***_54_ in *C. neoformans*. **(A)** The fungal cells were incubated with FITC-labeled Sparamosin_26–54_, and images were acquired using a confocal laser scanning microscopy. **(B)** Detection of Sparamosin_26–54_ binding to cellular lipids by protein-phospholipid interaction assay. **(C)** The densitometry analysis of PIP Strips^*TM*^ probed with Sparamosin_26–54_ by Image J software. Data represent mean ± standard error of mean from three independent biological replicates. Significant difference between blank group and cellular lipids group was indicated by asterisks as *p < 0.05; **p < 0.01; ***p < 0.001. LPA, lysophosphatidic acid; LPC, lysophosphocholine; PI, phosphatidylinositol; PI(3)P, phosphatidylinositol (3)-phosphate; PI(4)P, phosphatidylinositol (4)-phosphate; PI(5)P, phosphatidylinositol (5)-phosphate; PE, phosphatidylethanolamine; PC, phosphatidylcholine; S1P, sphingosine 1-phosphate; PI(3,4)P_2_, phosphatidylinositol (3,4)-bisphosphate; PI(3,5)P_2_, phosphatidylinositol (3,5)-bisphosphate; PI(4,5)P_2_, phosphatidylinositol (4,5)-bisphosphate; PI(3,4,5)P_3_, phosphatidylinositol (3,4,5)-trisphosphate; PA, phosphatidic acid; PS, phosphatidylserine.

In many cases, binding to the surface of fungal cell is the first step for AMPs to kill fungi. Therefore, we hypothesized that Sparamosin_26_***_–_***_54_ might directly bind to the cell surface of *C*. *neoformans*. To test this hypothesis, the distribution of Sparamosin_26_***_–_***_54_ in *C*. *neoformans* was investigated by FITC-labeled Sparamosin_26_***_–_***_54_. As shown in Figure 4A, the fluorescence of FITC-labeled Sparamosin_26_***_–_***_54_ was mainly located on the cell surface, indicating that Sparamosin_26_***_–_***_54_ might interact with the cell walls/membranes. To further determine the binding properties of Sparamosin_26_***_–_***_54_ to cell wall or cell membrane components, a modified ELISA assay and chitin-binding assay were performed. The results showed that Sparamosin_26_***_–_***_54_ could not bind glucan or chitin (data not shown). Next, we assessed the ability of Sparamosin_26_***_–_***_54_ to bind different bioactive membrane phospholipids using the protein-phospholipid interaction assay. As shown in Figures 4B,C, Sparamosin_26_***_–_***_54_ strongly bound to phosphoinositides (PIPs) and phosphatidic acid (PA), but weakly bound to a variety of other phospholipids, including lysophosphatidic acid (LPA), lysophosphocholine (LPC), phosphatidylinositol (PI), phosphatidylethanolamine (PE), phosphatidylcholine (PC), sphingosine 1-phosphate (S1P), and phosphatidylserine (PS). These results suggest that Sparamosin_26_***_–_***_54_ might bind to fungal cell membrane rather than cell wall.

**FIGURE 5:**
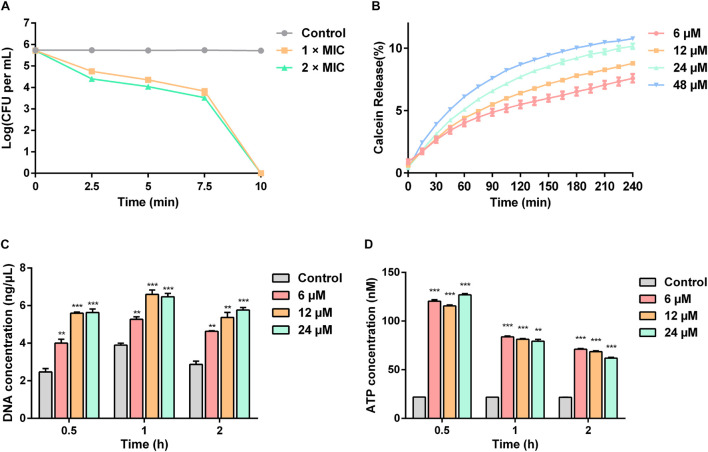
Sparamosin_26–54_ kills *C*. *neoformans* via disrupting the integrity of cell membranes. **(A)** The time-killing curves of Sparamosin_26–54_ against *C. neoformans*. **(B)** The time course of calcein release from *C. neoformans* induced by Sparamosin_26–54_. **(C)** The concentration of extracellular DNA in *C. neoformans* after Sparamosin_26–54_ treatment. The amount of extracellular DNA in the supernatants was quantified by spectrophotometry at 260 nm. **(D)** The concentration of extracellular ATP in *C. neoformans* after Sparamosin_26–54_ treatment. The amount of extracellular ATP was detected using the ATP determination kit. Data represent mean ± standard error of mean from three independent biological replicates. Significant difference between control group and Sparamosin_26–54_ treatment group was indicated by asterisks as **p < 0.01; ***p < 0.001.

**FIGURE 6:**
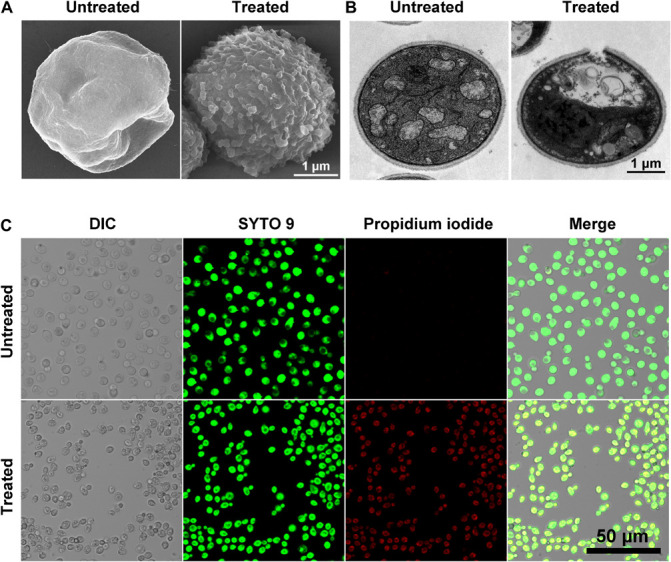
Scanning electron microscope (SEM), transmission electron microscope (TEM) and confocal laser scanning microscopy (CLSM) images show the effect of Sparamosin_26–54_ on *C. neoformans*. **(A,B)** The morphology of *C. neoformans* treated with Sparamosin_26–54_ observed by SEM and TEM. **(C)** Live-dead staining assay shows the differentially stained cells after Sparamosin_26–54_ treatment.

The time-killing curves for Sparamosin_26_***_–_***_54_ on *C*. *neoformans* was determined at the concentrations of 1 × and 2 × MIC. As shown in Figure 5A, Sparamosin_26_***_–_***_54_ showed rapid killing activity against *C. neoformans* and completely killed the fungus within 10 min. This rapid fungicidal rate indicated that Sparamosin_26_***_–_***_54_ might kill *C. neoformans* by disrupting the integrity of cell membrane. To further evaluate the effect of Sparamosin_26_***_–_***_54_ on the permeability of the *C*. *neoformans* cell membrane, the release of calcein, DNA and ATP were measured. As shown in Figure 5B, when exposed to Sparamosin_26_***_–_***_54_, calcein leaked from calcein-loaded *C*. *neoformans* in a time- and concentration-dependent manner. In addition, Sparamosin_26_***_–_***_54_ increased the amount of DNA and ATP detected in the supernatant of the *C*. *neoformans* suspensions (Figures 5C,D). Then, we employed SEM and TEM to observe the changes in the morphology and ultrastructure of *C*. *neoformans* cells after Sparamosin_26_***_–_***_54_ treatment (Figures 6A,B). The SEM images of the peptide-treated cells showed that Sparamosin_26_***_–_***_54_ had a morbid effect on *C*. *neoformans* cells surface. After treatment with 24 μM Sparamosin_26_***_–_***_54_ for 1 h, the surface of the fungal cells became rough and corrugated. In contrast, the control cells that were not treated with Sparamosin_26_***_–_***_54_ exhibited a bright and smooth surface. The TEM image of the peptide-treated cells clearly showed the disruption of cell wall and cell membrane and the leakage of intracellular contents, while the fungal cells in the control group showed an intact cell wall and cell membrane and a homogeneous cytoplasm. SYTO 9 can enter all yeasts regardless of their membrane integrity, while PI can only enter yeasts with damaged membranes. Therefore, the integrity of the *C*. *neoformans* cell membrane was observed by staining with SYTO 9/PI. As shown in Figure 6C, the untreated cells were stained only with SYTO 9 instead of PI, while cells treated with Sparamosin_26_***_–_***_54_ were stained with SYTO 9 and PI. Taken together, these results suggested that Sparamosin_26_***_–_***_54_ disrupts the integrity of the cell wall and cell membrane of *C*. *neoformans*.

### Sparamosin_26–54_ Induces Apoptosis in *C. neoformans*

**FIGURE 7:**
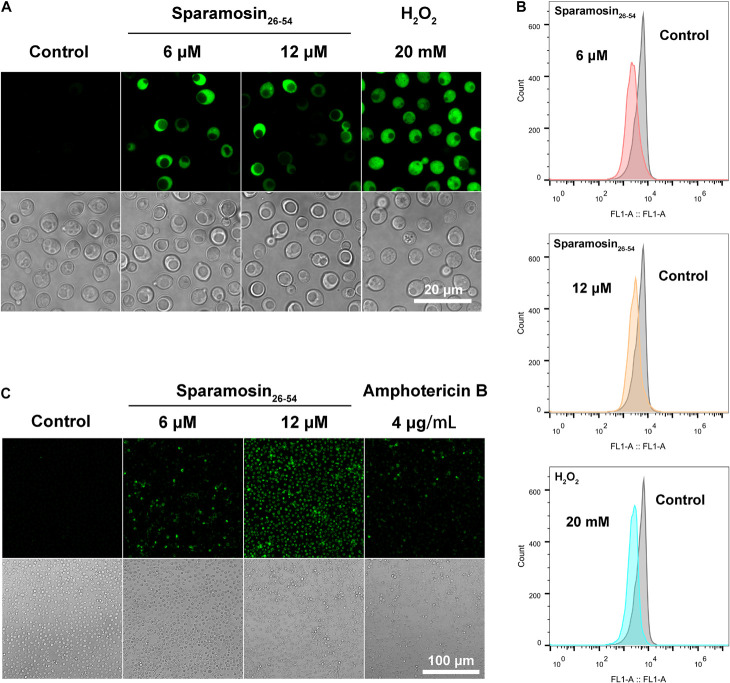
Sparamosin_26–54_ induces apoptosis in *C. neoformans*. **(A)** Detection of intracellular ROS accumulation in Sparamosin_26–54_-treated *C. neoformans* using DCFH-DA. **(B)** Analysis of mitochondrial membrane depolarization in *C. neoformans* after Sparamosin_26–54_ treatment. **(C)** Analysis of DNA damage in Sparamosin_26–54_-treated *C. neoformans* by the TUNEL assay.

Among the DEGs identified, a gene coding for a metacaspase was found to be up-regulated under Sparamosin_26_***_–_***_54_ treatment, indicating that Sparamosin_26_***_–_***_54_ may induce apoptosis in *C*. *neoformans*. Thus, we first used DCFH-DA probe to measure intracellular ROS in *C. neoformans* during exposure to Sparamosin_26_***_–_***_54_. As shown in Figure 7A, cells treated with 20 mM H_2_O_2_ as a positive control showed increased DCH staining compared to untreated cells. In accordance with this, cells exposed to Sparamosin_26_***_–_***_54_ (6 and 12 μM) displayed a significant increase in intracellular ROS levels compared to untreated cells. These data indicated that Sparamosin_26_***_–_***_54_ treatment resulted in the accumulation of ROS in *C. neoformans*. The dissipation of MMP is considered to be an early and key cellular event in the occurrence of apoptosis (Barroso et al., 2006). To investigate mitochondria-mediated pathway during apoptosis in *C. neoformans* induced by Sparamosin_26_***_–_***_54_, we next examined MMP of *C. neoformans* by using DiOC_6_(3) which is a membrane potential-sensitive dye that aggregating in healthy mitochondria. After treatment with 6 and 12 μM Sparamosin_26_***_–_***_54_ for 3 h, MMP was observed by the decrease of the fluorescence intensity of DiOC_6_(3), indicating that Sparamosin_26_***_–_***_54_ disrupted the MMP of *C. neoformans* (Figure 7B). To obtain more evidence of apoptosis in *C. neoformans* induced by Sparamosin_26_***_–_***_54_, the TUNEL assay was conducted, which is one of the most reliable methods for identifying the late stage of yeast cell apoptosis, and is used to visualize the amount of DNA fragmentation in individual cells. We used amphotericin B as a positive control because it has been proven to induce apoptosis in *C. neoformans* cells (Castro et al., 2018). The microscopic images showed the presence of green fluorescence in the cells treated with 6 and 12 μM Sparamosin_26_***_–_***_54_, which was consistent with the positive control cells treated with 4 μg/mL amphotericin B (Figure 7C). Since accumulation, MMP dissipation and DNA fragmentation are all hallmarks of apoptosis (Munoz et al., 2012), our data collectively confirmed that *C. neoformans* treated with Sparamosin_26_***_–_***_54_ exhibited apoptosis mechanism.

## Discussion

*C*. *neoformans* is an important fungal pathogen, causing life-threatening pneumonia and meningitis in immunocompromised patients. However, the existing treatment options for cryptococcosis have been hampered by their inherent toxicity to humans and the increase in their drug-resistance. This situation urgently requires the development of novel antifungal agents. Due to the broad-spectrum antimicrobial activities and unique mode of action against human pathogens, natural AMPs have attracted more and more attention as potential alternatives for conventional antibiotics. To date, over 3,000 natural AMPs have been found (antimicrobial peptide database 3, APD3) (Wang et al., 2016). In the past few decades, dozens of AMPs have been identified from the mud crab *S*. *paramamosain*, such as crustins, anti-lipopolysaccharide factors, and several gonadal-specific AMPs including scygonadin, SCY2 and scyreprocin (Huang et al., 2006; Wang et al., 2007; Qiao et al., 2016; Wang et al., 2018; Yang et al., 2020; Long et al., 2021). In the study, we identified a novel AMP named Sparamosin from *S*. *paramamosain*, and found that its truncated peptide Sparamosin_26_***_–_***_54_ showed broad-spectrum antimicrobial activity. Furthermore, Sparamosin_26_***_–_***_54_ exerts potent inhibitory effect against the three infection forms of *C. neoformans* (that is, planktonic cells, biofilm formation and preformed biofilm). It has multiple antifungal mechanisms against *C. neoformans*, including disruption of the cell wall and cell membrane integrity and induction of apoptosis. Transcriptome analysis showed that after Sparamosin_26_***_–_***_54_ treatment, the expression of genes related to cell wall component biosynthesis, CWI signaling pathway, anti-oxidative stress, apoptosis, DNA repair, ergosterol biosynthesis pathway and mitochondrial oxidative phosphorylation were significantly modulated in *C. neoformans*. These obtained results provide important reference for the further development of Sparamosin_26_***_–_***_54_ as a new antifungal drug.

Sequence modification is an effective strategy to improve the performance of natural AMPs. Various sequence modification methods attempted to modify natural AMPs by deleting, adding, or replacing one or more amino acid residues, truncating peptides, or assembling chimeric peptides from fragments of different natural AMPs. Many AMPs, such as cecropins, LL-37, magainins, and melittins, are finally obtained through sequence modification (Oh et al., 2000; Wu et al., 2014; Wang et al., 2019). Due to the synthetic mature peptide of Sparamosin exerts moderate inhibitory effect against bacteria and fungi, two truncated peptides, Sparamosin_1_***_–_***_25_ and Sparamosin_26_***_–_***_54_, were designed and synthesized based on the secondary structure and physicochemical parameters of Sparamosin. Through predicting the secondary structure of Sparamosin, we found that there were two α-helices located at residues Ile^13^-Phe^24^ and residues Pro^31^-Arg^39^, respectively. The α-helical AMPs, such as cecropin, magainin and LL-37, are one of the most widely studied AMPs (Beevers and Dixon, 2010; Nguyen et al., 2011). Many of these α-helical AMPs are usually cationic and amphipathic with potent antimicrobial activities against bacteria and fungi (Hancock and Sahl, 2006). The mechanism of action of α-helical AMPs is mainly composed of two steps, that is, the initial binding of cationic AMPs with the negatively charged components on the microbial cell surface and the subsequent membrane disruption (Huang, 2000; Beevers and Dixon, 2010). The crucial physicochemical parameters of peptides were calculated by using online tools. In this study, the hydrophobic residue ratio of Sparamosin and its truncated peptides ranged from 32 to 38%, and the net charge varied from −3 to + 6. Hydrophobicity is defined as the percentage of hydrophobic residues in the peptide and is an important parameter to determine the activity of AMPs. The hydrophobicity exceeds a threshold to confer antimicrobial activity, and there is an upper limit, beyond which the host cells will rupture and cell selectivity will be lost (Frederiksen et al., 2020). Therefore, the percentage of hydrophobic residues in AMPs characterized to date is approximately 40–60% (Tossi et al., 2000). Cationicity is another important parameter that affects the antimicrobial activity of AMPs. Most AMPs are positively charged, and charges from + 4 to + 6 seem to be the most common (Tossi et al., 2000). Within a certain range, an increase in the total charge of cationic peptides will often lead to an increase in affinity for microbial membranes, thereby enhancing their antimicrobial activity (Dathe et al., 2001). It should be noted that hydrophobicity and cationicity are not independent, they complement each other and together determine the antimicrobial activity of AMPs. Among Sparamosin and its truncated peptides, Sparamosin_1_***_–_***_25_ has no antimicrobial activities against the tested strains, and Sparamosin_26_***_–_***_54_ displayed stronger antimicrobial activity than Sparamosin. Compared with Sparamosin, the hydrophobic residue ratio of the Sparamosin_26_***_–_***_54_ increased from 35 to 38%, and the net charge increased from + 3 to + 6, which might the main reason why its activity is stronger than Sparamosin. The underlying mechanism still need further evidence.

It is noteworthy that Sparamosin_26_***_–_***_54_ also showed excellent anti-biofilm activity against *C. neoformans*. In the present study, we found that Sparamosin_26_***_–_***_54_ could effectively prevent the formation of biofilm, presumably due to the reduction of the planktonic population, and cause the damage to the growth of preformed biofilms through a strong inhibition of respiration. A biofilm is an organized aggregate of microbial cells that attached to a solid surface and enclosed in an extracellular polymeric substance matrix (Donlan, 2002). *C. neoformans* is a yeast-like fungus with polysaccharide capsules that can form biofilms on polystyrene plates and medical devices (Martinez and Casadevall, 2005). The biofilm formation of *C. neoformans* includes fungal surface adhesion, microcolony formation, and matrix production (Martinez and Casadevall, 2005). Fungal cells within biofilms display unique phenotypic characteristics that can increase the resistance to the host’s immune system and antifungal agents (Lilit et al., 2017; Kean and Ramage, 2019). Azole drugs such as voriconazole and fluconazole can only effectively inhibit planktonic fungal cells, but have little effect on fungal biofilms (Martinez and Casadevall, 2006). Amphotericin B and caspofungin are the two most effective drugs for preventing the formation of *C. neoformans* biofilm and against mature biofilms (Martinez and Casadevall, 2006). However, the effective concentration of amphotericin B and caspofungin against *C. neoformans* biofilm is higher than the therapeutic range, thus causing serious toxicity (Martinez and Casadevall, 2006; Delattin et al., 2014). In fact, most of the currently available antibiotics are unable to address chronic infections caused by biofilm effectively (Roy et al., 2018; Vuotto and Donelli, 2019). In addition to broad-spectrum bactericidal and fungicidal activities, more and more evidences showed that AMPs can also exhibit anti-biofilm activity against both bacterial and fungal biofilm in three different ways by reducing planktonic population, preventing cells from initially adhering to the surface, and eradicating established biofilms. For example, a shortened variant of mouse cathelicidin-related AMP, termed AS10, inhibits the formation of *C*. *albicans* biofilms, and acts synergistically with common antifungal drugs (such as amphotericin B and caspofungin) against mature biofilms (De Brucker et al., 2014). The mud crab AMP Scyreprocin could not only inhibit the biofilm formation, but also eradicate mature biofilms of *C. albicans* and *C. neoformans* (Yang et al., 2020). Therefore, the research and development of AMPs with anti-biofilm activity has more clinical significance. The anti-biofilm mechanism of Sparamosin_26_***_–_***_54_ and the efficacy of Sparamosin_26_***_–_***_54_ combined with standard antifungal drugs is worthy of further study.

The proposed antifungal mechanisms of AMPs are diverse, including cell wall integrity disruption, cell membrane permeabilization as well as apoptosis induction (Hwang et al., 2011b; Cho et al., 2012; Ma et al., 2020). Many studies have also shown that AMPs and pore-forming toxins have a similar mechanism of action, that is, the formation of holes in the cell membrane (Gilbert et al., 2014; Etxaniz et al., 2018). It has been reported that pore-forming toxins could trigger very diverse response pathways in eukaryotic cells (Gonzalez et al., 2011; Kao et al., 2011). When the plasma membrane is damaged by pore-forming toxins, cells activate signaling pathways to restore plasma membrane integrity and ion homeostasis (Gonzalez et al., 2011). In addition, pore-forming toxins can induce cell death by inducing apoptosis and pyrophosis (Genestier et al., 2005; Shoma et al., 2008). To investigate the antifungal mechanism of Sparamosin_26_***_–_***_54_, we used RNA-Seq to study the transcriptomic profile of *C. neoformans* treated with sublethal concentrations of Sparamosin_26_***_–_***_54_. As reported previously, sublethal concentrations of AMPs can lead to fungal cell dysfunction. For example, the plant defensin HsAFP1 at 2 × FC50 (FC50, fungicidal concentration resulting in 50% killing) induces autophagy, vacuolar dysfunction and cell cycle impairment in *C. albican* (Struyfs et al., 2020). The *Musca domestica* antifungal peptide-1 (MAF-1) at 1 × MIC induces the expression of genes related to oxidative stress response and cell wall synthesis and inhibits the expression of genes related to metabolism and fatty acid biosynthesis in *C. albican* (Wang et al., 2017). In the study, *C. neoformans* cells were incubated with different concentrations of Sparamosin_26_***_–_***_54_ (0.25 × MIC, 0.5 × MIC) for 1 h before RNA sequencing. We compared the differential gene-expression profile of Sparamosin_26_***_–_***_54_ treatment at 0.25 × MIC and 0.5 × MIC to the untreated control to identify a set of genes commonly perturbed by Sparamosin_26_***_–_***_54_ treatment. This analysis revealed that genes involved in the biosynthesis of cell wall component, CWI signaling pathway, anti-oxidative stress, apoptosis and DNA repair were upregulated under Sparamosin_26_***_–_***_54_ treatment, while genes associated with the ergosterol biosynthesis pathway and mitochondrial oxidative phosphorylation were down-regulated under the same condition. It is not clear which genes or signaling pathways are specifically involved in the cellular response to Sparamosin_26_***_–_***_54_. The use of inhibitors or regulators of specific signaling pathways to demonstrate the involvement of pathways in the cell death process deserves further investigation.

We propose that cellular changes and damage initiated through interaction with primary targets of Sparamosin_26_***_–_***_54_ result in activation of intracellular signaling pathways. Fungal cell membrane is potential target for AMPs and is rich in a variety of lipids such as glycerophospholipids, sphingolipids and sterols. In this study, we identified the glycerophospholipid targets of Sparamosin_26_***_–_***_54_ as PIPs and PA. In addition, the rapid fungicidal effect of Sparamosin_26_***_–_***_54_ against *C. neoformans* was associated with increased plasma membrane permeability, which can be detected by releasing calcein from fungal cells, leaking DNA and ATP to the supernatant, and positively staining yeasts with PI, and observed by SEM and TEM. Thus, it could be speculated that the binding of Sparamosin_26_***_–_***_54_ to PIPs and PA induces the membrane permeability of *C. neoformans*. PIPs are usually found on the inner leaflet of eukaryotic cells and play a major role in many important membrane-related processes, such as signal transduction, ion channel function, endocytosis, exocytosis, etc. (McLaughlin and Murray, 2005). Although PIPs commonly exist in eukaryotic cells, the relative contributions of negative and neutral glycerophospholipids in cell membrane of fungi and mammal are different (Rautenbach et al., 2016). Generally, fungal cell membranes are mainly composed of PI and PS, which tend to be high electronegative, while mammalian cell membranes rich in zwitterionic phospholipids PC are usually neutral in net charge (Rautenbach et al., 2016). These electrostatic differences between fungi and mammalian cells may allow a stronger initial electrostatic interaction between the cationic AMPs and the fungal cell surface. Certain plant defensins have been observed to bind PIPs preferentially. For example, the *Nicotiana alata* defensin NaD1 could bind to a wide range of PIPs, while the tomato defensin TPP3 specifically bound to PI(4,5)P_2_ (Baxter et al., 2015; Payne et al., 2016). The binding of NaD1 to PI(4,5)P_2_ leads to the formation of an oligomeric complex, that is critical for cytolytic activity (Poon et al., 2014). This unique peptide-lipid interaction was common in plant defensins, which may be one of the important mechanisms of plants against fungal infection. Interestingly, we found that AMPs from marine animal and plant have the similar binding properties to membrane phospholipid, suggesting that Sparamosin_26_***_–_***_54_ and plant defensins have similar mechanisms of action. Further studies are needed to understand the role of phospholipids binding in Sparamosin_26_***_–_***_54_-induced membrane permeabilization of *C. neoformans*.

In addition to the membrane disruption, some AMPs can also induce oxidative stress, DNA damage and apoptosis in yeasts (Cho and Lee, 2011; Hwang et al., 2011a, b). ROS, such as hydrogen peroxide (H_2_O_2_), hydroxyl radicals (OH⋅) and superoxide anions (O_2_^–^), are considered to be early signal mediators of apoptosis (Fleury et al., 2002). The ROS produced by aerobic metabolism usually exist in cells in balance with antioxidant enzymes (such as catalase, superoxide dismutase, and glutathione peroxidase) (Dantas et al., 2015). Previous studies have shown that the increase in ROS upon AMPs treatment also occurs in fungi, which supports our findings (Hwang et al., 2011a; Wang et al., 2015). Excessive ROS has multiple deleterious effects on the essential structures of fungi (such as nucleic acid, DNA, amino acid residues, and cell membrane) resulting in cell death (Perrone et al., 2008). Mitochondria play an important role in energy conversion, cell signaling and apoptosis pathway (Turrens, 2003). The cell permeability and lipophilic dye, DiOC_6_(3), which accumulates in healthy mitochondria and is widely used to investigate the mitochondria-mediated pathways during apoptosis (Zamzami et al., 1996; Kataoka et al., 2005). Our present study demonstrated that after treatment with Sparamosin_26_***_–_***_54_, DiOC_6_(3) dye no longer accumulated in mitochondria, resulting in a decrease in green fluorescence, suggesting that Sparamosin_26_***_–_***_54_ might lead to the opening of mitochondrial membrane transition pores and induce the dissipation of the MMP. The dysfunction of mitochondria leads to an energy crisis and promotes the release of proapoptotic factors from mitochondria into the cytosol, which then activates caspase (Heiskanen et al., 1999; Pereira et al., 2007). During apoptosis, chromatin DNA is cut into small fragments by the activation of endonucleases, which is an irreversible step of apoptosis. The labeling and observation of DNA fragmentation by TUNEL assay is one of the most reliable methods to identify the phenotype of late apoptosis in yeasts (Ribeiro et al., 2006). Taken together, our comprehensive physiological effect data (including ROS accumulation, MMP dissipation and DNA fragmentation) indicated that Sparamosin_26_***_–_***_54_ induced apoptosis in *C. neoformans*.

In summary, the present study demonstrated the antifungal activity and related mechanism of Sparamosin_26_***_–_***_54_, which is a novel AMP found in mud crab *S*. *paramamosain*. The synthetic Sparamosin_26_***_–_***_54_ showed potent activity against three infection forms of *C. neoformans* (planktonic, biofilm formation and preformed biofilm). It was confirmed that this peptide can effectively kill *C. neoformans* via multiple modes of action, including disrupting the integrity of the cell wall and cell membrane and inducing apoptosis. These results indicated that the novel AMP Sparamosin_26–54_ is expect to be a promising antifungal drug that could be used to control *C. neoformans* infection in the future.

## Data Availability Statement

The datasets presented in this study can be found in online repositories. The names of the repository/repositories and accession number(s) can be found below: https://www.ncbi.nlm.nih.gov/, PRJNA751241. The GenBank accession number is MH423837.

## Author Contributions

K-JW and FC: conceptualization, funding acquisition, project administration, supervision, and writing—review and editing. Y-CC: data curation, formal analysis, investigation, methodology, and writing—original draft. YY, CZ, and H-YC: investigation and methodology. All authors contributed to the article and approved the submitted version.

## Conflict of Interest

The authors declare that the research was conducted in the absence of any commercial or financial relationships that could be construed as a potential conflict of interest.

## Publisher’s Note

All claims expressed in this article are solely those of the authors and do not necessarily represent those of their affiliated organizations, or those of the publisher, the editors and the reviewers. Any product that may be evaluated in this article, or claim that may be made by its manufacturer, is not guaranteed or endorsed by the publisher.

## Footnotes

1
http://www.cbs.dtu.dk/services/SignalP/

2
http://bioinf.cs.ucl.ac.uk/psipred/

3
https://web.expasy.org/protparam/

4
https://aps.unmc.edu/

## References

[B1] Barroso G. Taylor S. Morshedi M. Manzur F. Oehninger S. (2006). Mitochondrial membrane potential integrity and plasma membrane translocation of phosphatidylserine as early apoptotic markers: a comparison of two different sperm subpopulations. *Fertil. Steril.* 85 149–154. 10.1016/j.fertnstert.2005.06.046 16412746

[B2] Baxter A. A. Richter V. Lay F. T. Poon I. K. Adda C. G. Veneer P. K. (2015). The tomato defensin TPP3 binds phosphatidylinositol (4, 5)-bisphosphate via a conserved dimeric cationic grip conformation to mediate cell lysis. *Mol. Cell. Biol.* 35 1964–1978. 10.1128/MCB.00282-15 25802281 PMC4420927

[B3] Beevers A. J. Dixon A. M. (2010). Helical membrane peptides to modulate cell function. *Chem. Soc. Rev.* 39 2146–2157. 10.1039/b912944h 20502803

[B4] Berditsch M. Jger T. Strempel N. Schwartz T. Ulrich A. S. (2015). Synergistic effect of membrane-active peptides polymyxin B and gramicidin S on multidrug-resistant strains and biofilms of *Pseudomonas aeruginosa*. *Antimicrob. Agents Chemother.* 59:5288. 10.1128/AAC.00682-15 26077259 PMC4538509

[B5] Brogden K. A. (2005). Antimicrobial peptides: pore formers or metabolic inhibitors in bacteria? *Nat. Rev. Microbiol.* 3 238–250. 10.1038/nrmicro1098 15703760

[B6] Brown G. D. Denning D. W. Gow N. A. Levitz S. M. Netea M. G. White T. C. (2012). Hidden killers: human fungal infections. *Sci. Transl. Med.* 4:165rv113. 10.1126/scitranslmed.3004404 23253612

[B7] Campoy S. Adrio J. L. (2017). Antifungals. *Biochem. Pharmacol.* 133 86–96. 10.1016/j.bcp.2016.11.019 27884742

[B8] Castro S. C. D. Taissa V. Sonia R. Kelly I. (2018). Miltefosine has a postantifungal effect and induces apoptosis in *Cryptococcus yeasts*. *Antimicrob. Agents Chemother.* 62:e00312-18. 10.1128/AAC.00312-18 29844051 PMC6105859

[B9] Chen B. Fan D.-Q. Zhu K.-X. Shan Z.-G. Chen F.-Y. Hou L. (2015). Mechanism study on a new antimicrobial peptide Sphistin derived from the N-terminus of crab histone H2A identified in haemolymphs of *Scylla paramamosain*. *Fish Shellfish Immunol.* 47 833–846. 10.1016/j.fsi.2015.10.010 26475366

[B10] Chen H. M. Chan S. C. Lee J. C. Chang C. C. Murugan M. Jack R. W. (2003). Transmission electron microscopic observations of membrane effects of antibiotic cecropin B on *Escherichia coli*. *Microsc. Res. Tech.* 62 423–430. 10.1002/jemt.10406 14601148

[B11] Cho J. Hwang I.-S. Choi H. Hwang J. H. Hwang J.-S. Lee D. G. (2012). The novel biological action of antimicrobial peptides via apoptosis induction. *J. Microbiol. Biotechnol.* 22 1457–1466. 10.4014/jmb.1205.05041 23124334

[B12] Cho J. Lee D. G. (2011). Oxidative stress by antimicrobial peptide pleurocidin triggers apoptosis in *Candida albicans*. *Biochimie* 93 1873–1879. 21782000 10.1016/j.biochi.2011.07.011

[B13] Dantas A. D. S. Day A. Ikeh M. Kos I. Achan B. Quinn J. (2015). Oxidative stress responses in the human fungal pathogen, *Candida albicans*. *Biomolecules* 5 142–165. 10.3390/biom5010142 25723552 PMC4384116

[B14] Dathe M. Nikolenko H. Meyer J. Beyermann M. Bienert M. (2001). Optimization of the antimicrobial activity of magainin peptides by modification of charge. *FEBS Lett.* 501 146–150. 10.1016/s0014-5793(01)02648-5 11470274

[B15] Datta A. Yadav V. Ghosh A. Choi J. Bhattacharyya D. Kar R. K. (2016). Mode of action of a designed antimicrobial peptide: high potency against *Cryptococcus neoformans*. *Biophys. J.* 111 1724–1737. 10.1016/j.bpj.2016.08.032 27760359 PMC5071555

[B16] De Brucker K. Delattin N. Robijns S. Steenackers H. Verstraeten N. Landuyt B. (2014). Derivatives of the mouse cathelicidin-related antimicrobial peptide (CRAMP) inhibit fungal and bacterial biofilm formation. *Antimicrob. Agents Chemother.* 58 5395–5404. 10.1128/AAC.03045-14 24982087 PMC4135870

[B17] Delattin N. Cammue B. P. Thevissen K. (2014). Reactive oxygen species-inducing antifungal agents and their activity against fungal biofilms. *Future Med. Chem.* 6 77–90. 10.4155/fmc.13.189 24358949

[B18] Denning D. W. Bromley M. J. (2015). Infectious Disease. How to bolster the antifungal pipeline. *Science* 347 1414–1416. 10.1126/science.aaa6097 25814567

[B19] Destoumieux D. Bulet P. Loew D. Van Dorsselaer A. Rodriguez J. Bachere E. (1997). Penaeidins, a new family of antimicrobial peptides isolated from the shrimp *Penaeus vannamei* (Decapoda). *J. Biol. Chem.* 272 28398–28406. 10.1074/jbc.272.45.28398 9353298

[B20] Destoumieux D. Bulet P. Strub J. M. van Dorsselaer A. Bachère E. (1999). Recombinant expression and range of activity of penaeidins, antimicrobial peptides from penaeid shrimp. *Eur. J. Biochem.* 266 335–346. 10.1046/j.1432-1327.1999.00855.x 10561573

[B21] Donlan R. M. (2002). Biofilms: microbial life on surfaces. *Emerg. Infect. Dis.* 8:881. 10.3201/eid0809.020063 12194761 PMC2732559

[B22] Dutcher J. D. William G. Pagano J. F. John V. (1959). *Amphotericin b, Its Production, and Its Salts.* US patent application 2,908,611. Washington, DC: US Environmental Protection Agency.

[B23] Ellis D. (2002). Amphotericin B: spectrum and resistance. *J. Antimicrob. Chemother.* 49(Suppl._1), 7–10. 10.1093/jac/49.suppl_1.7 11801575

[B24] Etxaniz A. González-Bullón D. Martín C. Ostolaza H. (2018). Membrane repair mechanisms against permeabilization by pore-forming toxins. *Toxins* 10:234. 10.3390/toxins10060234 29890730 PMC6024578

[B25] Fanos V. Cataldi L. (2000). Amphotericin B-induced nephrotoxicity: a review. *J. Chemother.* 12 463–470. 10.1179/joc.2000.12.6.463 11154026

[B26] Felix B. Sara G. Rita O. David D. (2017). Global and multi-national prevalence of fungal diseases-estimate precision. *J. Fungi* 3:57. 10.3390/jof3040057 29371573 PMC5753159

[B27] Fleury C. Mignotte B. Vayssière J. (2002). Mitochondrial reactive oxygen species in cell death signaling. *Biochimie* 84 131–141. 10.1016/s0300-9084(02)01369-x 12022944

[B28] Frederiksen N. Hansen P. R. Zabicka D. Tomczak M. Urbas M. Domraceva I. (2020). Alternating cationic-hydrophobic peptide/peptoid hybrids: influence of hydrophobicity on antibacterial activity and cell selectivity. *ChemMedChem* 15 2544–2561. 10.1002/cmdc.202000526 33029927

[B29] Genestier A. L. Michallet M. C. Prévost G. Bellot G. Chalabreysse L. Peyrol S. (2005). Staphylococcus aureus panton-valentine leukocidin directly targets mitochondria and induces Bax-independent apoptosis of human neutrophils. *J. Clin. Invest.* 115 3117–3127. 10.1172/jci22684 16276417 PMC1265849

[B30] Gilbert R. J. Dalla Serra M. Froelich C. J. Wallace M. I. Anderluh G. (2014). Membrane pore formation at protein-lipid interfaces. *Trends Biochem. Sci.* 39 510–516. 10.1016/j.tibs.2014.09.002 25440714

[B31] Gonzalez M. R. Bischofberger M. Frêche B. Ho S. Parton R. G. van der Goot F. G. (2011). Pore-forming toxins induce multiple cellular responses promoting survival. *Cell Microbiol.* 13 1026–1043. 10.1111/j.1462-5822.2011.01600.x 21518219

[B32] Gudmundsson G. H. Agerberth B. Odeberg J. Bergman T. Olsson B. Salcedo R. (1996). The human gene FALL39 and processing of the cathelin precursor to the antibacterial peptide LL-37 in granulocytes. *Eur. J. Biochem.* 238 325–332. 10.1111/j.1432-1033.1996.0325z.x 8681941

[B33] Gueguen Y. Garnier J. Robert L. Lefranc M.-P. Mougenot I. De Lorgeril J. (2006). PenBase, the shrimp antimicrobial peptide penaeidin database: sequence-based classification and recommended nomenclature. *Dev. Comp. Immunol.* 30 283–288. 10.1016/j.dci.2005.04.003 15963564

[B34] Hancock R. E. Sahl H.-G. (2006). Antimicrobial and host-defense peptides as new anti-infective therapeutic strategies. *Nat. Biotechnol.* 24 1551–1557. 10.1038/nbt1267 17160061

[B35] Heiskanen K. M. Bhat M. B. Wang H.-W. Ma J. Nieminen A.-L. (1999). Mitochondrial depolarization accompanies cytochrome c release during apoptosis in PC6 cells. *J. Biol. Chem.* 274 5654–5658. 10.1074/jbc.274.9.5654 10026183

[B36] Huang H. W. (2000). Action of antimicrobial peptides: two-state model. *Biochemistry* 39 8347–8352. 10.1021/bi000946l 10913240

[B37] Huang W. S. Wang K. J. Yang M. Cai J. J. Li S. J. Wang G. Z. (2006). Purification and part characterization of a novel antibacterial protein Scygonadin, isolated from the seminal plasma of mud crab, *Scylla serrata* (Forskål, 1775). *J. Exp. Mar. Biol. Ecol.* 339 37–42. 10.1016/j.jembe.2006.06.029

[B38] Hwang B. Hwang J.-S. Lee J. Lee D. G. (2011b). The antimicrobial peptide, psacotheasin induces reactive oxygen species and triggers apoptosis in *Candida albicans*. *Biochem. Biophys. Res. Commun.* 405 267–271. 10.1016/j.bbrc.2011.01.026 21219857

[B39] Hwang B. Hwang J.-S. Lee J. Kim J.-K. Kim S. R. Kim Y. (2011a). Induction of yeast apoptosis by an antimicrobial peptide, Papiliocin. *Biochem. Biophys. Res. Commun.* 408 89–93. 10.1016/j.bbrc.2011.03.125 21458420

[B40] Iyer K. R. Revie N. M. Fu C. Robbins N. Cowen L. E. (2021). Treatment strategies for cryptococcal infection: challenges, advances and future outlook. *Nat. Rev. Microbiol.* 19 454–466. 10.1038/s41579-021-00511-0 33558691 PMC7868659

[B41] Kao C. Y. Los F. C. Huffman D. L. Wachi S. Kloft N. Husmann M. (2011). Global functional analyses of cellular responses to pore-forming toxins. *PLoS Pathog.* 7:e1001314. 10.1371/journal.ppat.1001314 21408619 PMC3048360

[B42] Kataoka M. Fukura Y. Shinohara Y. Baba Y. (2005). Analysis of mitochondrial membrane potential in the cells by microchip flow cytometry. *Electrophoresis* 26 3025–3031. 10.1002/elps.200410402 16078196

[B43] Kean R. Ramage G. (2019). Combined antifungal resistance and biofilm tolerance: the global threat of *Candida auris*. *mSphere* 4:e00458-19. 10.1128/mSphere.00458-19 31366705 PMC6669339

[B44] Lemke A. Kiderlen A. Kayser O. (2005). Amphotericin b. *Appl. Microbiol. Biotechnol.* 68 151–162. 10.1007/s00253-005-1955-9 15821914

[B45] Lilit A. David S. Silvana V. Eliseo E. Raddy R. Luis M. (2017). The crucial role of biofilms in Cryptococcus neoformans survival within macrophages and colonization of the central nervoussystem. *J. Fungi Open Access Mycol. J.* 3:10. 10.3390/jof3010010 29371529 PMC5715963

[B46] Liu H.-P. Chen R.-Y. Zhang Q.-X. Wang Q.-Y. Li C.-R. Peng H. (2012). Characterization of two isoforms of antiliopolysacchride factors (Sp-ALFs) from the mud crab *Scylla paramamosain*. *Fish Shellfish Immunol.* 33 1–10. 10.1016/j.fsi.2012.03.014 22538350

[B47] Liu J. Chen F. Wang X. Peng H. Zhang H. Wang K.-J. (2020). The synergistic effect of mud crab antimicrobial peptides Sphistin and Sph12-38 with antibiotics azithromycin and rifampicin enhances bactericidal activity against *Pseudomonas aeruginosa*. *Front. Cell. Infect. Microbiol.* 10:e572849. 10.3389/fcimb.2020.572849 33194811 PMC7645104

[B48] Livak K. J. Schmittgen T. D. (2001). Analysis of relative gene expression data using real-time quantitative PCR and the 2-ΔΔCT method. *Methods* 25 402–408. 10.1006/meth.2001.1262 11846609

[B49] Long S. Chen F. Wang K.-J. (2021). Characterization of a new homologous anti-lipopolysaccharide factor SpALF7 in mud crab *Scylla paramamosain*. *Aquaculture* 534:736333. 10.1016/j.aquaculture.2020.736333

[B50] Loyse A. Burry J. Cohn J. Ford N. Chiller T. Ribeiro I. (2019). Leave no one behind: response to new evidence and guidelines for the management of cryptococcal meningitis in low-income and middle-income countries. *Lancet Infect. Dis.* 19 e143–e147. 10.1016/S1473-3099(18)30493-6 30344084

[B51] Ma H. Zhao X. Yang L. Su P. Fu P. Peng J. (2020). Antimicrobial peptide AMP-17 affects *Candida albicans* by disrupting its cell wall and cell membrane integrity. *Infect. Drug Resist.* 13:2509. 10.2147/IDR.S250278 32801789 PMC7398874

[B52] Martinez L. R. Casadevall A. (2005). Specific antibody can prevent fungal biofilm formation and this effect correlates with protective efficacy. *Infect. Immun.* 73 6350–6362. 10.1128/IAI.73.10.6350-6362.2005 16177306 PMC1230912

[B53] Martinez L. R. Casadevall A. (2006). Susceptibility of Cryptococcus neoformans biofilms to antifungal agents in vitro. *Antimicrob. Agents Chemother.* 50 1021–1033. 10.1128/AAC.50.3.1021-1033.2006 16495265 PMC1426450

[B54] McLaughlin S. Murray D. (2005). Plasma membrane phosphoinositide organization by protein electrostatics. *Nature* 438 605–611. 10.1038/nature04398 16319880

[B55] Miceli M. H. Díaz J. A. Lee S. A. (2011). Emerging opportunistic yeast infections. *Lancet Infect. Dis.* 11 142–151. 10.1016/S1473-3099(10)70218-8 21272794

[B56] Munoz A. J. Wanichthanarak K. Meza E. Petranovic D. (2012). Systems biology of yeast cell death. *FEMS Yeast Res.* 12 249–265. 10.1111/j.1567-1364.2011.00781.x 22188402

[B57] Nguyen L. T. Haney E. F. Vogel H. J. (2011). The expanding scope of antimicrobial peptide structures and their modes of action. *Trends Biotechnol.* 29 464–472. 10.1016/j.tibtech.2011.05.001 21680034

[B58] Oh D. Shin S. Y. Lee S. Kang J. H. Kim S. D. Ryu P. D. (2000). Role of the hinge region and the tryptophan residue in the synthetic antimicrobial peptides, cecropin A(1-8)-magainin 2(1-12) and its analogues, on their antibiotic activities and structures. *Biochemistry* 39 11855–11864. 10.1021/bi000453g 11009597

[B59] Pappas P. G. Alexander B. D. Andes D. R. Hadley S. Kauffman C. A. Freifeld A. (2010). Invasive fungal infections among organ transplant recipients: results of the transplant-associated infection surveillance network (TRANSNET). *Clin. Infect. Dis.* 50 1101–1111. 10.1086/651262 20218876

[B60] Payne J. A. Bleackley M. R. Lee T.-H. Shafee T. M. Poon I. K. Hulett M. D. (2016). The plant defensin NaD1 introduces membrane disorder through a specific interaction with the lipid, phosphatidylinositol 4, 5 bisphosphate. *Biochim. Biophys. Acta Biomembr.* 1858 1099–1109. 10.1016/j.bbamem.2016.02.016 26896695

[B61] Pereira C. Camougrand N. Manon S. Sousa M. J. Côrte-Real M. (2007). ADP/ATP carrier is required for mitochondrial outer membrane permeabilization and cytochrome c release in yeast apoptosis. *Mol. Microbiol.* 66 571–582. 10.1111/j.1365-2958.2007.05926.x 17822411

[B62] Perfect J. R. (2017). The antifungal pipeline: a reality check. *Nat. Rev. Drug Discov.* 16 603–616. 10.1038/nrd.2017.46 28496146 PMC5760994

[B63] Perlin D. S. Rautemaa-Richardson R. Alastruey-Izquierdo A. (2017). The global problem of antifungal resistance: prevalence, mechanisms, and management. *Lancet Infect. Dis.* 17 e383–e392. 10.1016/S1473-3099(17)30316-X 28774698

[B64] Perrone G. G. Tan S.-X. Dawes I. W. (2008). Reactive oxygen species and yeast apoptosis. *Biochim. Biophys. Acta Mol. Cell Res.* 1783 1354–1368. 10.1016/j.bbamcr.2008.01.023 18298957

[B65] Peschel A. Sahl H.-G. (2006). The co-evolution of host cationic antimicrobial peptides and microbial resistance. *Nat. Rev. Microbiol.* 4 529–536. 10.1038/nrmicro1441 16778838

[B66] Poon I. K. Baxter A. A. Lay F. T. Mills G. D. Adda C. G. Payne J. A. (2014). Phosphoinositide-mediated oligomerization of a defensin induces cell lysis. *eLife* 3:e01808. 10.7554/eLife.01808 24692446 PMC3968744

[B67] Poonam K. Rutusmita M. Neha A. Apurva C. Rashmi G. Partha R. (2017). Antifungal and anti-biofilm activity of essential oil active components against Cryptococcus neoformans and *Cryptococcus laurentii*. *Front. Microbiol.* 8:2161. 10.3389/fmicb.2017.02161 29163441 PMC5681911

[B68] Qiao K. Xu W.-F. Chen H.-Y. Peng H. Zhang Y.-Q. Huang W.-S. (2016). A new antimicrobial peptide SCY2 identified in *Scylla paramamosain* exerting a potential role of reproductive immunity. *Fish Shellfish Immunol.* 51 251–262. 10.1016/j.fsi.2016.02.022 26911409

[B69] Rajasingham R. Smith R. M. Park B. J. Jarvis J. N. Govender N. P. Chiller T. M. (2017). Global burden of disease of HIV-associated cryptococcal meningitis: an updated analysis. *Lancet Infect. Dis.* 17 873–881. 10.1016/S1473-3099(17)30243-8 28483415 PMC5818156

[B70] Rautenbach M. Troskie A. M. Vosloo J. A. (2016). Antifungal peptides: to be or not to be membrane active. *Biochimie* 130 132–145. 10.1016/j.biochi.2016.05.013 27234616

[B71] Ribeiro G. F. Côrte-Real M. Johansson B. (2006). Characterization of DNA damage in yeast apoptosis induced by hydrogen peroxide, acetic acid, and hyperosmotic shock. *Mol. Biol. Cell* 17 4584–4591. 10.1091/mbc.e06-05-0475 16899507 PMC1635349

[B72] Rowley A. F. Powell A. (2007). Invertebrate immune systems-specific, quasi-specific, or nonspecific? *J. Immunol.* 179 7209–7214. 10.4049/jimmunol.179.11.7209 18025161

[B73] Roy R. Tiwari M. Donelli G. Tiwari V. (2018). Strategies for combating bacterial biofilms: a focus on anti-biofilm agents and their mechanisms of action. *Virulence* 9 522–554. 10.1080/21505594.2017.1313372 28362216 PMC5955472

[B74] Shan Z. Zhu K. Peng H. Chen B. Liu J. Chen F. (2016). The new antimicrobial peptide SpHyastatin from the mud crab *Scylla paramamosain* with multiple antimicrobial mechanisms and high effect on bacterial infection. *Front. Microbiol.* 7:1140. 10.3389/fmicb.2016.01140 27493644 PMC4954822

[B75] Shockey J. E. O’Leary N. A. de la Vega E. Browdy C. L. Baatz J. E. Gross P. S. (2009). The role of crustins in *Litopenaeus vannamei* in response to infection with shrimp pathogens: an in vivo approach. *Dev. Comp. Immunol.* 33 668–673. 10.1016/j.dci.2008.11.010 19100764

[B76] Shoma S. Tsuchiya K. Kawamura I. Nomura T. Hara H. Uchiyama R. (2008). Critical involvement of pneumolysin in production of interleukin-1alpha and caspase-1-dependent cytokines in infection with Streptococcus pneumoniae in vitro: a novel function of pneumolysin in caspase-1 activation. *Infect. Immun.* 76 1547–1557. 10.1128/iai.01269-07 18195026 PMC2292879

[B77] Smith V. J. Fernandes J. M. Kemp G. D. Hauton C. (2008). Crustins: enigmatic WAP domain-containing antibacterial proteins from crustaceans. *Dev. Comp. Immunol.* 32 758–772. 10.1016/j.dci.2007.12.002 18222540

[B78] Sperstad S. V. Haug T. Vasskog T. Stensvåg K. (2009b). Hyastatin, a glycine-rich multi-domain antimicrobial peptide isolated from the spider crab (*Hyas araneus*) hemocytes. *Mol. Immunol.* 46 2604–2612. 10.1016/j.molimm.2009.05.002 19487032

[B79] Sperstad S. V. Haug T. Paulsen V. Rode T. M. Strandskog G. Solem S. T. (2009a). Characterization of crustins from the hemocytes of the spider crab, *Hyas araneus*, and the red king crab, *Paralithodes camtschaticus*. *Dev. Comp. Immunol.* 33 583–591. 10.1016/j.dci.2008.10.010 19041340

[B80] Steiner H. Hultmark D. Engstrm H. H. B. Boman H. G. (1981). Sequence and specificity of two antibacterial proteins involved in insect immunity. *Nature* 292 246–248. 10.1038/292246a0 19454655

[B81] Struyfs C. Cools T. L. De Cremer K. Sampaio-Marques B. Ludovico P. Wasko B. M. (2020). The antifungal plant defensin HsAFP1 induces autophagy, vacuolar dysfunction and cell cycle impairment in yeast. *Biochim. Biophys. Acta Biomembr.* 1862:183255. 10.1016/j.bbamem.2020.183255 32145284 PMC7272304

[B82] Tossi A. Sandri L. Giangaspero A. (2000). Amphipathic, alpha-helical antimicrobial peptides. *Biopolymers* 55 4–30. 10931439 10.1002/1097-0282(2000)55:1<4::AID-BIP30>3.0.CO;2-M

[B83] Turrens J. F. (2003). Mitochondrial formation of reactive oxygen species. *J. Physiol.* 552 335–344. 14561818 10.1113/jphysiol.2003.049478 PMC2343396

[B84] Vitale R. G. Mouton J. W. Afeltra J. Meis J. F. Verweij P. E. (2002). Method for measuring postantifungal effect in *Aspergillus species*. *Antimicrob. Agents Chemother.* 46 1960–1965. 10.1128/AAC.46.6.1960-1965.2002 12019115 PMC127200

[B85] Vuotto C. Donelli G. (2019). Novel treatment strategies for biofilm-based infections. *Drugs* 79 1635–1655. 10.1007/s40265-019-01184-z 31468316

[B86] Vylkova S. Nayyar N. Li W. Edgerton M. (2007). Human β-defensins kill *Candida albicans* in an energy-dependent and salt-sensitive manner without causing membrane disruption. *Antimicrob. Agents Chemother.* 51 154–161. 10.1128/AAC.00478-06 17074797 PMC1797696

[B87] Wang G. Li X. Wang Z. (2016). APD3: the antimicrobial peptide database as a tool for research and education. *Nucleic Acids Res.* 44 1087–1093. 10.1093/nar/gkv1278 26602694 PMC4702905

[B88] Wang G. Narayana J. L. Mishra B. Zhang Y. Wang F. Wang C. (2019). Design of antimicrobial peptides: progress made with human cathelicidin LL-37. *Adv. Exp. Med. Biol.* 1117 215–240. 10.1007/978-981-13-3588-4_12 30980360

[B89] Wang H. Zhang J.-X. Wang Y. Fang W.-H. Wang Y. Zhou J.-F. (2018). Newly identified type II crustin (SpCrus2) in *Scylla paramamosain* contains a distinct cysteine distribution pattern exhibiting broad antimicrobial activity. *Dev. Comp. Immunol.* 84 1–13. 10.1016/j.dci.2018.01.021 29409789

[B90] Wang K. Dang W. Xie J. Zhu R. Sun M. Jia F. (2015). Antimicrobial peptide protonectin disturbs the membrane integrity and induces ROS production in yeast cells. *Biochim. Biophys. Acta Biomembr.* 1848 2365–2373. 10.1016/j.bbamem.2015.07.008 26209560

[B91] Wang K.-J. Huang W.-S. Yang M. Chen H.-Y. Bo J. Li S.-J. (2007). A male-specific expression gene, encodes a novel anionic antimicrobial peptide, scygonadin, in *Scylla serrata*. *Mol. Immunol.* 44 1961–1968. 10.1016/j.molimm.2006.09.036 17092560

[B92] Wang T. Xiu J. Zhang Y. Wu J. Ma X. Wang Y. (2017). Transcriptional responses of *Candida albicans* to antimicrobial peptide MAF-1A. *Front. Microbiol.* 8:894. 10.3389/fmicb.2017.00894 28567034 PMC5434131

[B93] Wiegand I. Hilpert K. Hancock R. E. (2008). Agar and broth dilution methods to determine the minimal inhibitory concentration (MIC) of antimicrobial substances. *Nat. Protoc.* 3:163. 10.1038/nprot.2007.521 18274517

[B94] Wu R. Wang Q. Zheng Z. Zhao L. Shang Y. Wei X. (2014). Design, characterization and expression of a novel hybrid peptides melittin (1-13)-LL37 (17-30). *Mol. Biol. Rep.* 41 4163–4169. 10.1007/s11033-013-2900-0 24871991

[B95] Yang Y. Chen F. Chen H.-Y. Peng H. Hao H. Wang K.-J. (2020). A novel antimicrobial peptide scyreprocin from mud crab *Scylla paramamosain* showing potent antifungal and anti-biofilm activity. *Front. Microbiol.* 11:1589. 10.3389/fmicb.2020.01589 32849331 PMC7396596

[B96] Yeung A. T. Gellatly S. L. Hancock R. E. (2011). Multifunctional cationic host defence peptides and their clinical applications. *Cell. Mol. Life Sci.* 68:2161. 10.1007/s00018-011-0710-x 21573784 PMC11114888

[B97] Zamzami N. Marchetti P. Castedo M. Hirsch T. Susin S. A. Masse B. (1996). Inhibitors of permeability transition interfere with the disruption of the mitochondrial transmembrane potential during apoptosis. *FEBS Lett.* 384 53–57. 10.1016/0014-5793(96)00280-3 8797802

[B98] Zasloff M. (1987). Magainins, a class of antimicrobial peptides from *Xenopus* skin: isolation, characterization of two active forms, and partial cDNA sequence of a precursor. *Proc. Natl. Acad. Sci. U.S.A.* 84 5449–5453. 10.1073/pnas.84.15.5449 3299384 PMC298875

[B99] Zasloff M. (2002). Antimicrobial peptides of multicellular organisms. *Nature* 415 389–395. 10.1038/415389a 11807545

[B100] Zhang R. Wang Z. Tian Y. Yin Q. Cheng X. Lian M. (2019). Efficacy of antimicrobial peptide DP7, designed by machine-learning method, against methicillin-resistant *Staphylococcus aureus*. *Front. Microbiol.* 10:1175. 10.3389/fmicb.2019.01175 31191493 PMC6546875

