# Supplementary material for: A Novel Antimicrobial Peptide Sparamosin26–54 From the Mud Crab Scylla paramamosain Showing Potent Antifungal Activity Against Cryptococcus neoformans
Source: Front Microbiol. 2021 Oct 8;12:746006. doi: 10.3389/fmicb.2021.746006 (PMC8531530; doi:10.3389/fmicb.2021.746006)
Supplement: Supplementary file 1 [file Data_Sheet_1.docx]

Supplementary Material

# Supplementary Figure


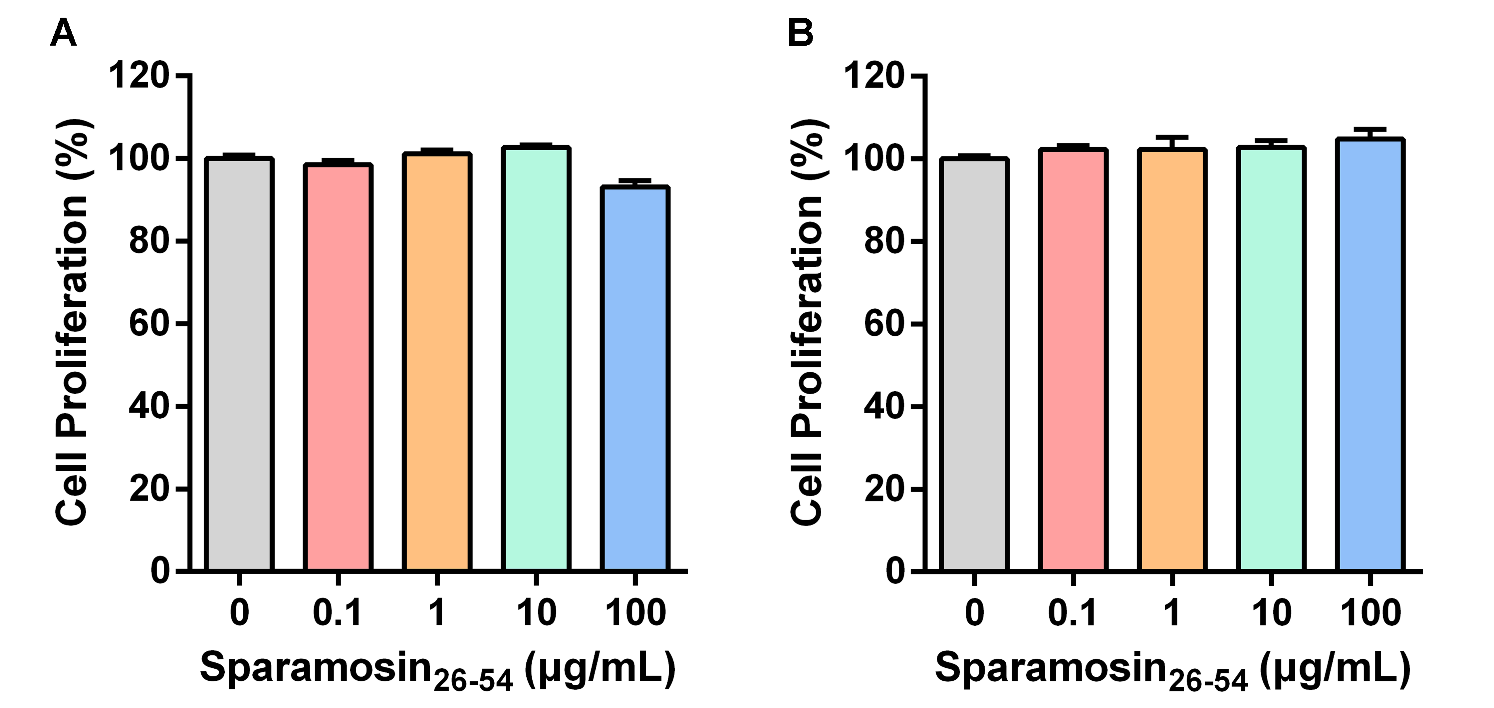


**Figure S1** Biocompatibility of Sparamosin_26-54_. **(A, B)** Cytotoxicity of Sparamosin_26-54_ against AML 12 cells **(A)** and L02 cells **(B)**. Data represent mean ± standard error of mean from three independent biological replicates.

# Supplementary Tables

**Table S1 The sequence amplification primers of Sparamosin.**

| **Primer name** | **Sequence 5′-3′** |
| --- | --- |
| Sparamosin-ORF-F | ATGGCGCGCCACGTGCTCCCG |
| Sparamosin-ORF-R | TCATCCAAACATACATGTGAA |
| Sparamosin 5'-1 | CCCAGGCCGCTGAAGATGGAATGTT |
| Sparamosin 5'-2 | TGAGTCGCACCACAAGAGCCACA |
| Sparamosin 3'-1 | TGTGGCTCTTGTGGTGCGACTCA |
| Sparamosin 3'-2 | TGTCTGCACCTGTCCCTGATCCA |
| Long primer^a^ | CTAATACGACTCACTATAGGGCAAGCAGTGGTATCAACGCAGAGT |
| Short primer^a^ | CTAATACGACTCACTATAGGGC |
| NUP^a^ | AAGCAGTGGTATCAACGCAGAGT |

^a^Long primer, short primer and NUP were provided by SMARTer™ RACE cDNA Amplification Kit.

**Table S2 The primers of reference gene and selected genes in qRT-PCR.**

| **Primer name** | **Sequence 5′-3′** |
| --- | --- |
| actin-F | ACAATGAGCTCCGTGTCGCT |
| actin-R | TCGGCCGGAAGCGTAAAGAG |
| CNA05300-F | TGAAGCGAGCGGATGTGAGG |
| CNA05300-R | CGTCACCTTGATGCGTTGCC |
| CNG02630-F | CGAGCTGGCGTTGTGGGATA |
| CNG02630-R | ATCCTTCTTGCACGCCACGA |
| CNF03720-F | ACCTCGAGAGGCCCTTGACT |
| CNF03720-R | TAGAGTTCTTGGGCGCGGTG |
| CNL06020-F | CAAGCGACGTTCAACCCTGC |
| CNL06020-R | AGCGGGTCTTCCTCCCTGAT |
| CND01070-F | AAGTACGCTGGTGAGCGAGC |
| CND01070-R | GGAAGTGACGGGGGCATCAA |
| CNA03530-F | AGCACCGACCTACGTCTCCT |
| CNA03530-R | TGGCGCTTCCCTTGAACCAT |
| CNL04470-F | CCAAGGCCGCCTATTACGGT |
| CNL04470-R | AAGCACCGGGGATCTTGTCG |
| CNK03240-F | CGTCACTTCCACCCGCTCAA |
| CNK03240-R | GGCAGCGACACCGACAAAGA |
| CNF02280-F | GGTAACCCCATCGACGGCAA |
| CNF02280-R | AAGCTCTCGCTGTCCACGAC |
| CND02890-F | GTGGCGCAGTTCAATCAGCC |
| CND02890-R | TTTGGCCATCACGCCAGACA |
| CNB00130-F | CTCACCCTCAGCATTTCGGT |
| CNB00130-R | GGACGTCGTTGATACAGCCA |

**Table S3 MIC of synthetic Sparamosin and its truncated peptides.**

| **Microbial strains** | **CGMCC**  **No.^a^** | **Sparamosin**  **MIC^b^ (μM)** | **Sparamosin_1-25_**  **MIC (μM)** | **Sparamosin_26-54_**  **MIC (μM)** |
| --- | --- | --- | --- | --- |
| **Gram-negative bacteria** |  |  |  |  |
| *Pseudomonas fluorescens* | 1.3202 | 12-24 | ＞48 | 3-6 |
| *Escherichia coli* | 1.2389 | 12-24 | ＞48 | 6-12 |
| **Gram-positive bacteria** |  |  |  |  |
| *Staphylococcus aureus* | 1.2465 | 12-24 | ＞48 | 6-12 |
| *Bacillus cereus* | 1.3760 | 24-48 | ＞48 | 6-12 |
| **Fungi** |  |  |  |  |
| *Cryptococcus neoformans* | 2.1563 | ＞48 | ＞48 | 6-12 |
| *Pichia pastoris* | - | 6-12 | ＞48 | 6-12 |

^a^CGMCC No., China General Microbiological Culture Collection Center Number.

^b^The values of MIC are expressed as the interval [a]-[b]. [a] is the highest concentration tested with visible microbial growth, while [b] is the lowest concentration without visible microbial growth.
